# Host plant specificity of the monarch butterfly *Danaus plexippus*: A systematic review and meta-analysis

**DOI:** 10.1371/journal.pone.0269701

**Published:** 2022-06-14

**Authors:** Lewis Greenstein, Christen Steele, Caz M. Taylor

**Affiliations:** 1 Illinois Natural History Survey, University of Illinois at Urbana-Champaign, Champaign, Illinois, United States of America; 2 Ecology and Evolutionary Biology, Tulane University, New Orleans, Louisiana, United States of America; University of Carthage, TUNISIA

## Abstract

The preference-performance hypothesis explains host specificity in phytophagous insects, positing that host plants chosen by adults confer the greatest larval fitness. However, adults sometimes oviposit on plants supporting low larval success because the components of host specificity (adult preference, plant palatability, and larval survival) are non-binary and not necessarily correlated. Palatability (willingness to eat) is governed by chemical cues and physical barriers such as trichomes, while survival (ability to complete development) depends upon nutrition and toxicity. Absence of a correlation between the components of host specificity results in low-performance hosts supporting limited larval development. Most studies of specificity focus on oviposition behavior leaving the importance and basis of palatability and survival under-explored. We conducted a comprehensive review of 127 plant species that have been claimed or tested to be hosts for the monarch butterfly *Danaus plexippus* to classify them as non-hosts, low performance, or high performance. We performed a meta-analysis to test if performance status could be explained by properties of neurotoxic cardenolides or trichome density. We also conducted a no-choice larval feeding experiment to identify causes of low performance. We identified 34 high performance, 42 low performance, 33 non-hosts, and 18 species with unsubstantiated claims. Mean cardenolide concentration was greater in high- than low-performance hosts and a significant predictor of host status, suggesting possible evolutionary trade-offs in monarch specialization. Other cardenolide properties and trichome density were not significant predictors of host status. In the experiment, we found, of the 62% of larvae that attempted to eat low-performance hosts, only 3.5% survived to adult compared to 85% of those on the high-performance host, demonstrating that multiple factors affect larval host plant specificity. Our study is the first to classify all known host plants for monarchs and has conservation implications for this threatened species.

## Introduction

Host-selection in phytophagous insects is a complex process mediated by many factors including oviposition stimulants [1, 2], feeding stimulants (phagostimulants) [3], vision [4], experience [5], and deterrents [6]. The preference-performance hypothesis explains the phenomenon of host specificity by positing that adult insects prefer to oviposit on plants that confer the greatest larval fitness [7–11] and most studies on host-specificity focus on adult oviposition behavior. For specialist insects, plant host status is often portrayed as binary (host or non-host) but, in reality, specificity is a multi-dimensional continuum including preference and performance. Performance can be further divided into palatability–larval willingness and ability to eat a plant–and survival–larval ability to develop into adulthood [5, 12, 13]. Palatability may be governed by chemical signals, i.e., phagostimulants and deterrents [14, 15], and physical barriers, such as trichomes and waxes [16, 17]. Survival is influenced by nutritional value and toxicity [18–20]. It has been shown that adult insects do not always select the host plants conferring the greatest larval fitness, demonstrating the importance of larval performance to fully understand host specificity [8–10, 21–25].

**Fig 1:**
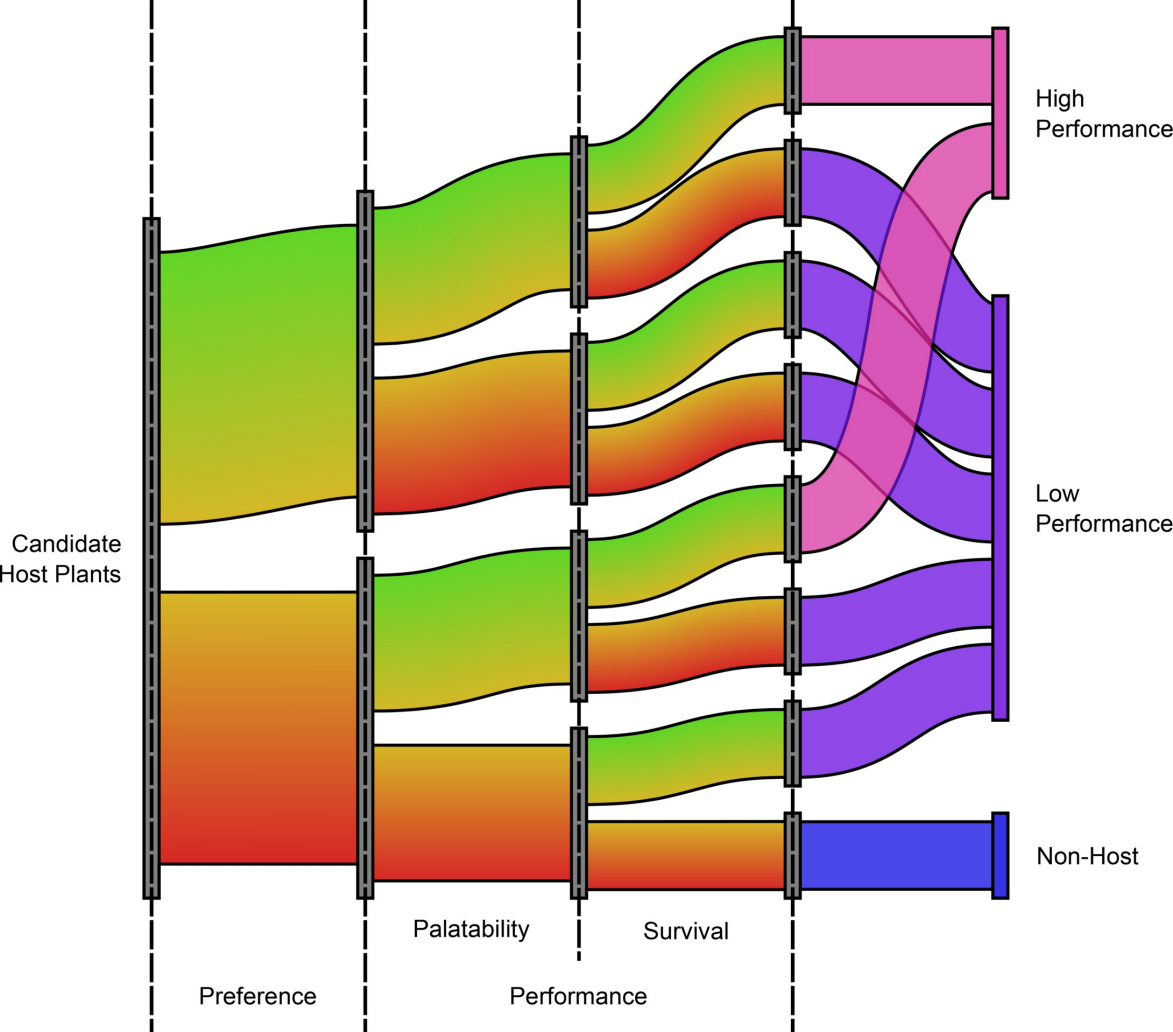
Components and continua of host status. Each of an insect’s potential host plants may land on different parts of preference, palatability, and survival continua, which are not necessarily correlated. A plant’s position along these continua determines its quality as a host.

The components of host specificity are encountered sequentially. Survival is irrelevant if palatability is too low to facilitate feeding and palatability is meaningless for plants on which adults do not oviposit. However, because all three components are continuous, “mistakes” in host selection are made, advancing a plant to the next component of host status, which may or may not also be low [13, 26]. It is possible for a low-preference plant to have high palatability and for a low-palatability plant to support high survival (Fig 1). These plants can help explain the biological and chemical factors underlying host selection and may be important to understanding shifts or expansions to new host plants [4, 27].

The monarch butterfly *Danaus plexippus* Linnaeus (*Lepidoptera*: *Nymphalidae*) is one of the best known examples of host specificity, as its larvae live and feed almost exclusively on milkweed plants in the genus *Asclepias* (*Apocynaceae*) [28]. Publications and online sources often mis-state true host status for monarchs, occasionally resulting in conflicting information. For example, the UK Natural History Museum’s HOSTS database [29] and the U.S. National Resources Conservation Service [30] list *Cynanchum laeve* (commonly climbing milkweed) as a monarch host plant but this species is absent from host lists published by the Xerces Society for Invertebrate Conservation [31], the U.S. Forest Service [32], and Monarch Joint Venture [33].

Even among *Asclepias* species, adult monarchs occasionally oviposit on plants that do not result in the greatest larval survival [5, 18, 34, 35]. This mismatch in oviposition preference and larval performance coupled with the inability of young larvae to move to better hosts necessitates research into the larval components of host specificity (palatability and survival). As with many other insects, considerable variation has been documented in monarch larval survival among host plants [5, 36–38] with differences of up to 45% between plants [18]. While some studies have reported mortality rates of larvae reared on uncommonly utilized plant species [34, 39], few attempts have been made to determine the drivers of variation in larval survival on these plants [6].

Cardenolides, also called cardiac glycosides (CGs), are part of the potential explanation for variation in monarch preference and performance. CGs are a class of steroid-derived toxins produced by many milkweeds and other plants [40, 41]. The CG backbone (genin) varies slightly, and each can have many different sugar moieties, resulting in a wide range of chemical properties, such as polarity. As a result, individual plants can produce a profile of many CG compounds [42–44]. Monarchs have evolved to contend with and even benefit from these chemicals in their host plants [45–50], but high concentrations of CGs can still decrease performance [51–55]. Additionally, less polar CGs and more diverse CG profiles tend to be more toxic [44, 55–60].

Physical and mechanical defenses may also contribute to the low palatability and survival conferred by some plants. Waxes and trichomes (small, hair-like structures covering many plants) act as physical barriers, slowing or preventing feeding. Young monarch larvae must ‘mow’ trichomes covering milkweed plants before they can begin feeding: a time-consuming process shown to reduce fitness [15, 57, 61–65]. Latex, the sticky sap exuded when milkweeds are damaged, reduces performance by miring larvae [52, 63] and also contains high levels of CGs [52, 56, 66].

Although monarchs have been extensively studied, the larval performance aspect of their host specificity is not well characterized, and there is conflicting information about which plants act as hosts. Monarch populations have declined in recent years, possibly due to scarcity of their milkweed hosts [67]. A deeper understanding of larval host specificity and identification of alternate host plants are therefore important to conservation efforts. To produce a comprehensive list of monarch host plants, we conducted a literature review of 127 potential host species and characterized what is known about their host status. We classified each plant as unsubstantiated, confirmed unpalatable, low performance, or high performance (Table 1). To investigate the importance of specific factors to palatability and survival in the differences between low- and high-performance hosts, we conducted a meta-analysis to test the hypotheses that plant trichome density, CG concentration, CG diversity, and CG polarity explain host status (A Table in S2 Appendix). Finally, we conducted a no-choice feeding experiment to evaluate the general, relative importance of palatability and survival to larval performance in a small number of species.

**Table 1:** Plant host classes and definitions.

Simplified Class	Class	Definition
Unsubstantiated	*U*	No evidence found that monarchs use this plant as a larval host; only unfounded claims.
	Unsubstantiated	
Non-Host	*N*	Larvae do not attempt to eat or take a bite but do not continue.
	Confirmed Unpalatable	
Low Performance	*L1*	Isolated observations of monarch larvae on these plants and/or these plants are oviposited on.
	Oviposit or Some Observations	
	*L2*	Larvae attempt to eat leaves but die prior to reaching adulthood.
	Lethal Attempt	
	*L3*	Larvae begin eating, but survival to adulthood has not been tested or ≤ 50% of larvae survive to adulthood.
	Some Attempt	
High Performance	*H1*	Many observations of larvae at different instars on these plants.
	Observed High Performance	
	*H2*	Larvae reared to adult on these plants, but no survival data is reported.
	Implied High Performance	
	*H3*	> 50% of larvae reared on these plants survive to adulthood.
	Tested High Performance	

## Materials and methods

### Literature review and classification

We first compiled a preliminary list of 76 monarch host plants from five online sources providing advice for non-scientists interested in monarch conservation [29–33]. Then, following the Preferred Reporting Items for Systematic reviews and Meta-Analyses (PRISMA) framework [68], we used Google Scholar to search the literature for evidence of an association between these species and monarchs (A Fig in S1 Appendix and A Fig in S2 Appendix). Synonymous scientific names were identified using the Global Biodiversity Information Facility (GBIF) database [69]. Searches were conducted with accepted scientific names, and publications using a different species name were noted. To reduce the likelihood of screening out relevant articles, relevant article citations were also assessed for inclusion. If the search returned no results, “Research-Grade” iNaturalist [70] observations of the plant were manually screened for co-observation with monarch larvae to account for potential publication bias. Sources containing information about associations between monarchs and plant species not on the preliminary list were added to the preliminary list to create a comprehensive list. The literature was further searched for associations with newly added plants using the previous methods. After duplicate studies were removed, all articles were manually screened for relevance based on publication and title. The full texts of remaining sources were manually screened to remove irrelevant sources (A Fig in S1 Appendix). Individual sources were assessed for bias, and notes may be found in A Table in S1 Appendix. We perceive the risk of bias in relevant, individual studies to be low and effectively managed because of our standardized protocol, broad search, inclusion of iNaturalist observations, and assessment of individual sources. This process in total produced a list of 127 possible host plant species.

Each plant on the list was classified as unsubstantiated (U), confirmed unpalatable (N), oviposit or some observations (L1), lethal attempt (L2), some attempt (L3), observed high performance (H1), implied high performance (H2) or confirmed high performance (H3) based on search results and iNaturalist observations as defined in Table 1. These classes were simplified into unsubstantiated (U), non-host (N), low performance (L1-L3), and high performance (H1-H3) for analysis (Table 1).

Complete methods, including PRISMA flow diagrams are available in S1 Appendix. A PRISMA Checklist is available in S1 File.

### Meta-analysis

Following the PRISMA framework, we used Google Scholar to search for aboveground CG concentrations, diversities, and polarities (A Fig in S2 Appendix) in addition to trichome densities (A Fig in S2 Appendix) for all low and high performance host plants on the list. We screened CG results, leaving only studies containing aboveground CG concentration measurements in units of (digitoxin equivalent mass)/(dry mass), analyses of polar plant extracts not finding cardenolides, CG diversity expressed as the Shannon-Wiener index applied to HPLC data [71], and the CG polarity index developed by Rasmann and Agrawal [71]. All aboveground CG concentrations excluding those from seeds were converted to units of (mg digitoxin equivalent)/(g of dry mass) (mg/g) and averaged for each plant species (grouping sub-species). Trichome search results were also filtered, leaving only publications indicating trichome density (# of trichomes/area) either on both abaxial and adaxial leaf surfaces or the sum of the abaxial and adaxial surfaces. Trichome data were converted to units of (# sum of both surfaces)/mm^2^ and averaged together for each species. Individual sources were assessed for bias, and notes may be found in B and C Tables in S2 Appendix. For this meta-analysis we draw only raw data from included sources because of a dearth of research evaluating the role of plant characteristics on host status at an inter-specific scale.

#### Statistical analysis

Average CG concentrations, CG polarities, CG diversities, and trichome densities were compared between high-performance and low-performance hosts using Wilcoxon rank sum tests because of the data’s non-normal distributions and inequal variances. We also calculate Hodges-Lehmann estimators (effect estimator) for significant Wilcoxon rank sum tests. To test the influence of plant characteristics (CG concentration, CG diversity, CG polarity, and trichome density) on plant host status, we constructed generalized linear models (GLMs) with binomial distributions using mean characteristic values for each plant species as predictor variables and low versus high performance status as a response. Because we were unable to find plant characteristic data for all species, we used three GLMs with different predictors to maximize sample sizes: CG concentration only, CG diversity and CG polarity, and trichome density only. Complete meta-analysis methods, including phylogenetic analyses and PRISMA resources, are included in S2 Appendix.

### No-choice feeding experiments

We performed no-choice feeding experiments to quantitatively compare the relative importance of palatability and survival on plant species with different performance statuses. We selected six plant species (in *Apocynaceae*, *Brassicaceae*, and *Solanaceae*) of varying palatability: three confirmed unpalatable plants (*Nerium oleander*, *Solanum dulacamara*, and *Brassica oleracea var*. *capitata*; commonly oleander, bittersweet nightshade, and cabbage respectively) as negative controls, two low-performance hosts, *Gonolobus suberosus* (anglepod) and *Araujia sericifera* (moth plant; both classified as L3), and one high-performance host, *Asclepias curassavica* (commonly tropical milkweed; classified as H3). All plants were purchased online or from local garden centers except *G*. *suberosus*, which was collected wild in the New Orleans, Louisiana area. Plants were watered *ad libitum* under greenhouse conditions.

Monarch butterflies were caught wild in New Orleans, Louisiana and first bred in captivity during October 2020. The first generation progeny of a single mating pair of adults (referred to as a family line) was used for each trial and each trial employed larvae from a different pair (A Table in S3 Appendix). Only adults that tested negative for the protozoan parasite *Ophryocystis elektroscirrha* were used for mating to reduce the probability of infecting larvae [72]. *O*. *elektroscirrha* is a detrimental parasite for monarch butterflies that can reduce the reproductive success and survival of larvae [73, 74]. Neonatal larvae (<24 hours old) were randomly assigned a feeding treatment before being placed in a petri dish with a moistened paper towel and an isolated piece of leaf tissue. Two larvae were placed in each dish with abundant food to eliminate the risk of competition. All experiments were carried out in an indoor laboratory where air temperature was maintained between 18 and 24°C.

After reaching the third instar, each larva was moved to a larger, individual enclosure with petioles of whole leaves or cut stems placed in water filled test tubes. To reduce exposure to bacteria and parasites, plant tissue was soaked in a 2% bleach solution for 2 minutes and then thoroughly rinsed with tap water before being given to larvae and was replaced as necessary due to wilting or consumption. The larval instar and presence of new frass was recorded daily for each individual until the larva died or entered the pupal stage. Experiments were carried out in four trials, and larvae from all but one trial were raised to adulthood (A Table in S3 Appendix).

#### Survival analysis

Larvae reared on non-host plants were pooled as negative controls for analysis. A Cox proportional hazard (CPH) model using species as a predictor variable in conjunction with a likelihood ratio *χ*^2^ test was used to determine if plant species was a significant predictor of survivorship curves.

To determine if host species was a significant predictor of differences in survival from days 1–5 and days 6–30, we used GLMs with larval survival during days 1–5 and from days 6–30 as response variables and plant species as a categorical predictor. All larvae reared on non-host plants (*B*. *oleracea var*. *capitata*, *N*. *oleander*, and *S*. *dulcamara*) died within 5 days of hatching and were excluded from both models. Individuals not reared to adulthood were excluded from the model of survival to day 30. Pairwise post-hoc tests (Tukey) were performed on the estimated marginal means for each species in these models to test for significant differences.

#### Development analysis

To identify differences in larval development time between plant species, we created a linear mixed model of development curves [75] with average larval instar on each day after hatching used as a response variable for the model and predictor variables of day after hatching and species (with negative controls pooled as non-hosts because these larvae did not develop; no other data were pooled for analysis). We included random effects of trial number and individual by day. Pairwise comparisons (Tukey) on the estimated marginal means of each species were carried out to test for differences between rearing treatments.

## Results

### Literature review

The host status of 127 plant species from 54 genera were assessed (B Table in S1 Appendix). We classified 18 as unsubstantiated (U), 33 as confirmed unpalatable (N), 5 as oviposit or some observations (L1), 8 as lethal attempt (L2), 29 as some attempt (L3), 8 as observed high performance (H1), 15 as implied high performance (H2), and 11 as confirmed high performance (H3). After simplification, 42 plants were identified as low-performance and 34 as high-performance hosts (Table 2). Our classification of 28 plant species conflicted with other lists of monarch host plants (excluding unsubstantiated claims) (B Table in S1 Appendix). No iNaturalist co-observations between monarchs and plants classified as unsubstantiated were found.

**Table 2:** Literature search results summary.

Data type	Total sources	Total species	High-performance species	Low-performance species
Host status	56	127	34	42
CG concentration	71	78	33	32
CG polarity	2	49	28	21
CG diversity	3	49	28	21
Trichome density	14	59	30	29

Full lists of included sources with notes and PRISMA resources are available in S1 and S2 Appendices. A PRISMA Checklist can be found in S1 File.

### Meta-analysis

#### Cardenolides

CG concentrations in aboveground tissues (excluding seeds) were identified for 78 plant species including 32 low- and 33 high-performance hosts from 71 unique sources (Table 2 and D Table in S2 Appendix). Mean cardenolide concentrations of aboveground plant tissues ranged from 0 mg/g (multiple species) to 7.2 mg/g (*Asclepias vestita*). Of the 41 sources that reported a sample size, the average was 17 plants. CG concentration for each species was obtained by averaging reported mean values from 1 to 29 sources (mean 3.86 ± 4.86 (sd) sources). The mean of standard deviations calculated between values for each plant species was 1.25 mg/g with values ranging from 0.03 (*A*. *pumila*) to 6.8 (*A*. *vestita*). We found sufficient evidence to include 11 plant species lacking cardenolides in analyses.

**Fig 2:**
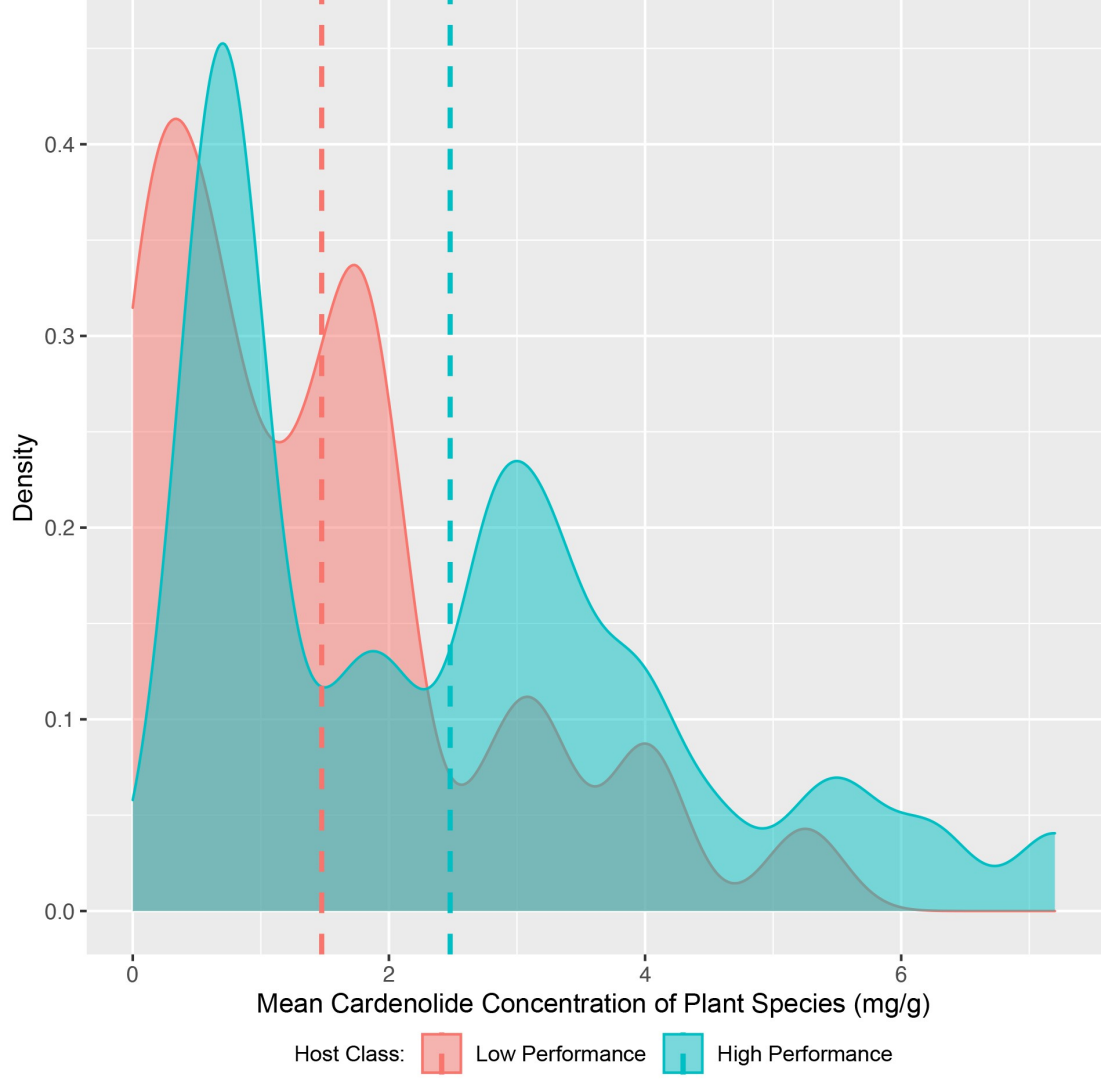
Density plot of plant species mean cardenolide concentrations. Vertical, dashed lines indicate mean values.

High-performance hosts had a greater average cardenolide concentration than low-performance hosts (*Z* = -2.64, p = 0.008) (Fig 2). The Hodges-Lehmann estimator was calculated as -0.72 mg/g (mean cardenolide concentration across all species = 1.67 ± 1.70 (sd) mg/g). Correspondingly, cardenolide concentration was a significant predictor of host status (*z*(63) = 2.67, p = 0.023) (G Table in S2 Appendix).

CG polarity and diversity values were found for 49 plant species, of which 21 were low performance and 28 were high performance (Table 2 and E Table in S2 Appendix). We found no significant differences in polarity or diversity between high- and low-performance plants (S2 Appendix). Neither polarity nor diversity were significant predictors of host status (H Table in S2 Appendix).

#### Trichome densities

We found trichome densities for 29 low-performance and 30 high-performance hosts (Table 2 and F Table in S2 Appendix). We found no significant differences in trichome densities between high- and low-performance host plants (S2 Appendix) and trichome density was not a significant predictor of host status (I Table in S2 Appendix).

When all non-peer reviewed sources (three conference papers and two academic theses; see A Table in S1 Appendix and B Table in S2 Appendix) were removed and the same statistical analyses were performed, our results remained identical (at the reported number of digits) to those presented above (S2 Appendix).

### No-choice feeding experiments

#### Survival

**Fig 3:**
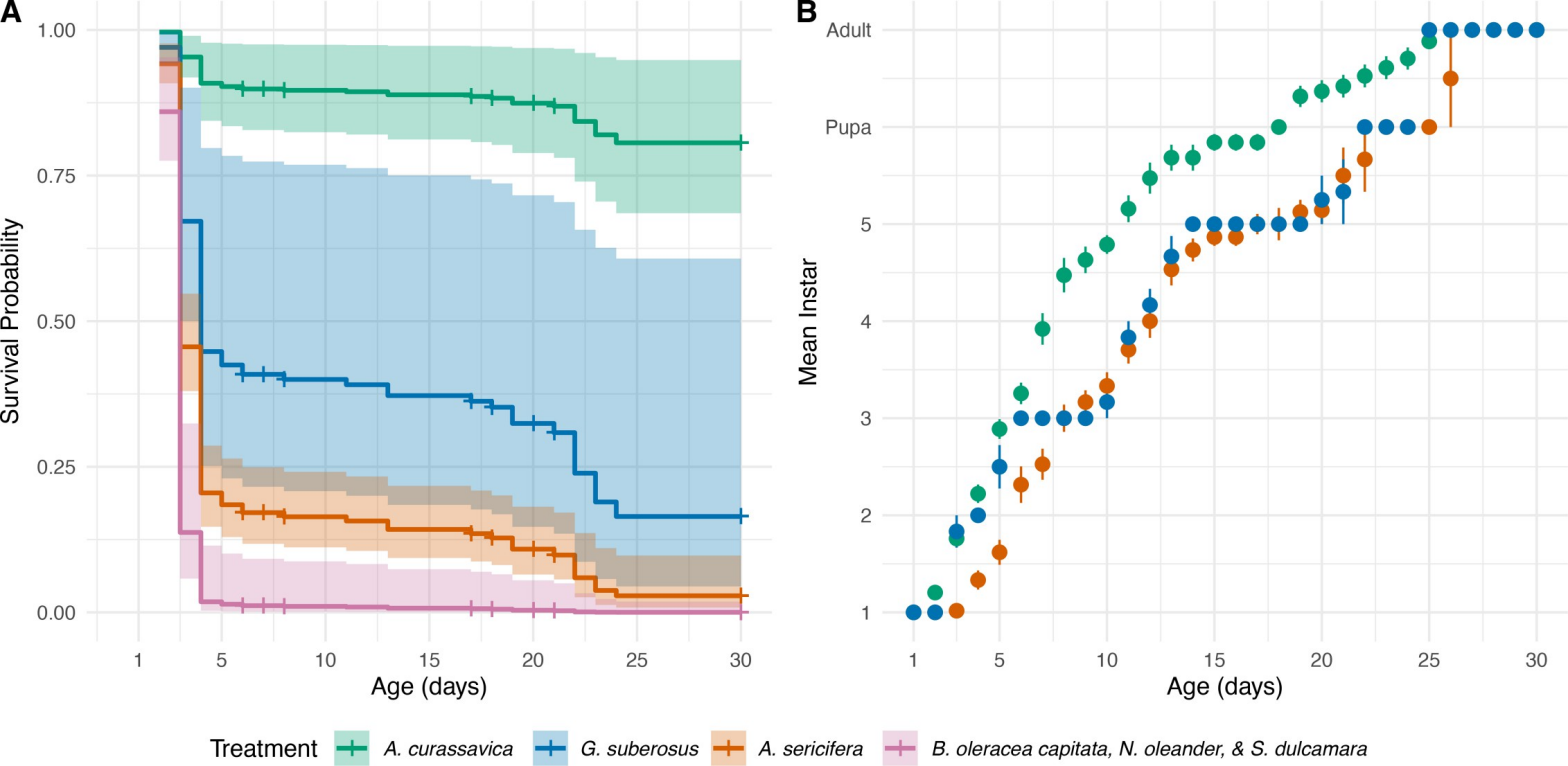
Kaplan Meyer curves from a CPH model and larval development over time. (A) Survivorship curves calculated by a CPH model from ages 1–30 days for larvae reared on different plant species. Negative controls (non-host species) were pooled. Color shaded areas represent 95% CIs of each curve. (B) The development of monarch larvae reared on different plants as the mean instar by larval age. Negative controls are omitted. Error bars indicate the standard error of mean instar for each day excluding dead individuals.

The Cox proportional hazard model was found to be globally significant when compared to the null model based on a likelihood ratio *χ*2 test (*χ*2(3) = 126.9, p = 2e-16) indicating that species or species group is a significant predictor of survivorship (B Table in S3 Appendix). The proportionality assumption for this model was upheld (*χ*2(3) = 1.38, p = 0.71). Examination of 95% confidence intervals showed that survivorship on the high-performance host, *Asclepias curassavica*, was significantly different than on non-hosts and low-performance hosts. Confidence intervals of the two low-performance hosts overlapped slightly, and therefore were not significantly different from each other (Fig 3A).

Two drops in survival probability (early die-off: days 1–5, late die-off: days 15–25) were observed in all plant treatments but varied in magnitude and cause (Fig 3A). Significantly larger early and late die-offs occurred when larvae were reared on *A*. *sericifera* than when they were reared on *A*. *curassavica*. The early die-off on *G*. *suberosus* was not significantly different from *A*. *curassavica* but was significantly larger than *A*. *sericifera*. However, the late die-off on *G*. *suberosus* was significantly larger than on *A*. *curassavica* and not significantly different from *A*. *sericifera* (Table 3).

**Table 3:** Pairwise Tukey post-hoc tests on the estimated marginal means of modeled larval survival from 1–5 and 6–30 days after hatching.

Plant Comparison	1–5 Day Survival				6–30 Day Survival			
	Estimate	SE	Z ratio	p value	Estimate	SE	Z ratio	p value
*A*. *curassavica—A*. *sericifera*	**4.56**	**1.053**	**4.333**	**<0.0001**	**4.391**	**1.13**	**3.882**	**0.0003**
*A*. *curassavica—G*. *suberosus*	2.25	1.246	1.807	0.1673	**3.750**	**1.38**	**2.721**	**0.0179**
*A*. *sericifera—G*. *suberosus*	**-2.31**	**0.746**	**-3.099**	**0.0055**	-0.642	1.32	-0.485	0.8786

Results are reported on the log odds ratio scale. All comparisons have infinite degrees of freedom. Model details can be found in C and D Tables in S3 Appendix. Comparisons where differences were statistically significantly different from zero are shown in bold.

**Fig 4:**
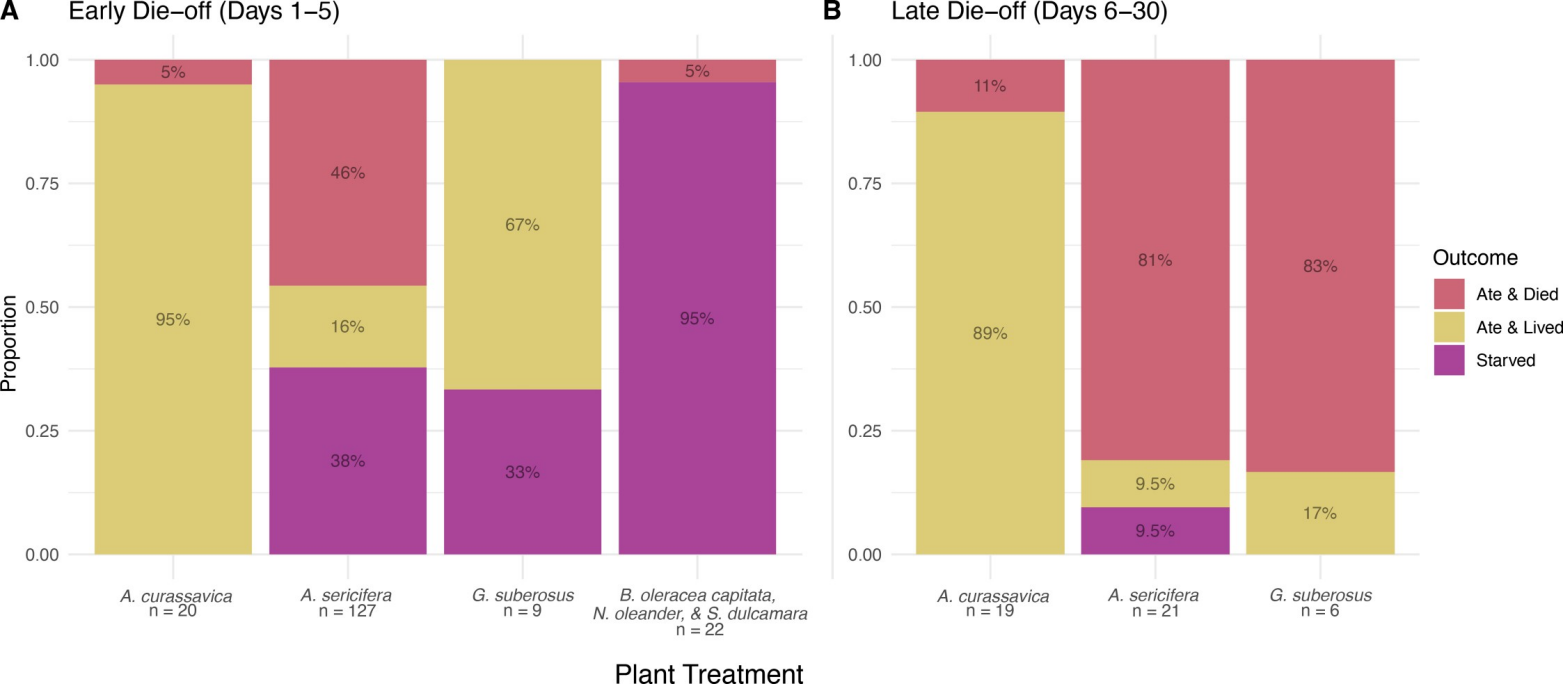
Outcomes of die-off events by plant treatment. (A) Outcomes of the early die-off. (B) Outcomes of the late die-off. Non-hosts are omitted because no larvae survived past day 5.

All 22 of the larvae reared on non-host plants (*B*. *oleracea var*. *capitata*, *N*. *oleander*, and *S*. *dulcamara*) died within 5 days of hatching, and only one individual produced frass (Fig 4A). Of the 127 larvae reared on *A*. *sericifera*, 2 (1.6%) reached adulthood, but 79 (63%) produced frass. *A*. *sericifera* larvae succumbing in the early die-off either did not attempt to eat or ate and still died, while 81% of the remaining individuals ate and died during the second die-off (Fig 4). Only 1 of 9 larvae (11%) reared on *G*. *suberosus* survived to adult, but 6 (67%) attempted to eat. *G*. *suberosus* larvae starved during the early die off and died despite eating during the late die-off (Fig 4). In contrast, 17 of 20 larvae (85%) reared on *A*. *curassavica* reached adult, despite all 20 producing frass.

#### Larval development

Larval development on *A*. *curassavica* was significantly faster than development on *A*. *sericifera* (*t*(134.8) = -10.551, p < 0.0001). The growth curve of larvae reared on *G*. *suberosus* was not significantly different from that of *A*. *curassavica* (*t*(90.7) = 2.089, p = 0.0979) or *A*. *sericifera* (*t*(101.4) = -2.343, p = 0.0544) (F Table in S3 Appendix).

## Discussion

The larval components of host specificity–plant palatability and larval survival–are influenced by numerous factors and are not necessarily correlated with each other resulting in a multi-dimensional continuum of host status. We identified a wide range of claims regarding the palatability of plants to monarch larvae that provided evidence for a broad list of possible host categories, blurring the distinction between what is a host and what is not. Our classification revealed some conflicting claims. Excluding unsubstantiated claims, we classified 28 plants differently from other sources (B Table in S1 Appendix). For example, some sources list *Euphorbia* and *Gossypium* as monarch host plants [29, 76], but these claims may be the result of mistranslation and misinterpretation of vernacular plant names [37]. *Citrus* and *Ipomea batatas* have also been documented as host plants [29]: a claim that appears to originate from observations of monarch larvae on these plants [77], but survival of larvae on these species has not been validated.

Another dimension on which to assess host status is the preference of adults to oviposit on a species, termed “oviposition use” [26]. The factors affecting oviposition behavior have been studied extensively. Multiple chemical oviposition stimulants have been identified [1, 2]. Taller plants and those with new growth are especially preferred by adults [55, 78, 79]. Oviposition use has even been shown to change depending on *O*. *elektroscirrha* infection status [80]. However, there do not appear to be differences between Eastern and Western monarch populations [35]. Some lists of monarch host plants ranked by oviposition preference exist [16, 39, 78, 79, 81–83], but these studies only include a few *Asclepias* species and, because adult oviposition behavior is affected by many environmental and contextual variables, wider comparisons of oviposition preference are difficult to make [5, 79, 81, 84, 85]. Further complicating the issue, oviposition use is a preference, meaning that pairwise comparisons between all plant species would be necessary to compile a comprehensive, ranked list. Future research is needed to further investigate the relationship between oviposition use and larval performance.

We found that cardenolide concentration was a significant and biologically relevant predictor of a plant species’ host class, suggesting a possible evolutionary tradeoff between high CG concentrations and more diverse cocktails of chemical defenses and its importance in the component of survival. Higher CG concentration was positively associated with performance despite previous research suggesting that milkweeds with higher cardenolide concentrations confer lower larval survival [52, 53, 55, 84]. Despite their toxicity, plants that produce cardenolides tend to produce fewer other defenses, potentially making these species better hosts [86–88]. Since milkweed plants produce other defensive compounds in addition to CGs [87, 89–92] and plants containing high CG concentrations tend to have lower CG inducibility and lower concentrations of non-CG defensive chemicals [86–88], it is possible that the evolution of cardenolide sequestration in monarchs occurred in part to avoid larval exposure to a more diverse cocktail of host plant defensive chemicals [93, 94].

It is possible that our cardenolide concentration analysis was confounded by genetic relatedness of plant species since we found no significant differences in CG concentration between low- and high-performance hosts using phylogenetic ANOVAs on the subset of species for which we had phylogenetic data. However, we also did not find any significant phylogenetic signal in mean cardenolide concentration or performance, and because the sample size was small, standard ANOVAs also did not reveal significant differences in CG concentration (S2 Appendix). A more comprehensive phylogeny would be needed to confirm our findings. Another potential factor affecting our results is that cardenolide concentration varies depending on multiple factors [51, 95–98]. However, we mitigated this risk by averaging together values from all available sources (3.86 on average) for each plant species.

Our experimental results support the roles of both palatability and survival in the classification of host status. During the early die-off, survival accounted for most larval mortality on *A*. *sericifera* as larvae attempted to eat this plant and produced frass, but low palatability nearly exclusively caused the mortality of larvae reared on *G*. *suberosus* and non-hosts. During the late die-off, mortality was almost entirely attributed to low survival (Fig 4). The different causes of mortality between die-offs in larvae reared on *A*. *sericifera* and *G*. *suberosus* demonstrate the potential for mismatch between the dimensions of performance. Pocius et al. [18] observed a similar, late die-off, which they partially attributed to variation in adult lipid content, supporting the importance of nutritional value to survival. Further experiments using full plants instead of excised leaves are necessary to further explore the role of latex in larval performance.

Despite controlling for larval genetics and environmental differences, we still observed considerable variation among individuals in feeding behavior and survival. There is no obvious explanation for why some larvae attempted to eat plants while others did not. It is possible that, because larval host status is determined by a combination of many factors, even full-sibling larvae have sufficient genetic variation across the many implicated loci to result in behavioral differences [99, 100] or are able to modulate gene expression differently [101]. Our results are unlikely to be affected by local adaptation. Freedman et al. [36] found that larval survival decreased from 79.7% to 75.7% when monarchs were reared on allopatric host plants, which is insufficient to justify geographic differences for host classes. Furthermore, even if the adults collected for experiments were part of the New Orleans resident population, they are unlikely to have adapted to local host plants (e.g., *A*. *curassavica* and *G*. *suberosus*) [102–104] because the population is young and gene flow with migratory populations is common [105–108].

Our classification may allow us to better characterize monarchs’ realized and fundamental host ranges [4, 109, 110]. For example, we found that *A*. *sericifera* supported development to adulthood albeit for a small number of larvae; however, adult monarchs rarely or never oviposit on this plant [78, 111]. This implies that *A*. *sericifera* is within the monarch fundamental host range, but outside of the realized host range. The identification of plant species that can support larval development but are not hosts in the wild introduces the possibility of host niche expansion in response to decreasing abundance of high-performance hosts, which could have important implications for conservation since the loss of *Asclepias* hosts has been blamed for declines in migratory monarch populations [67].

## Conclusion

Through a systematic review, we produced a comprehensive list of 34 high-performance and 42 low-performance host plants for monarch butterflies. We found that low performance can result from low palatability or low larval survival. Our meta-analysis showed that plants containing higher concentrations of cardenolides were more likely to be high-performance hosts, but other cardenolide properties and trichome density were not significant predictors of performance status. Further analyses and experiments are necessary to understand the specific causes of early and late larval mortality in addition to the cause of variation in feeding behavior among closely related individuals. The results of this study suggest the possibility of host range expansion and could be useful in monarch conservation as a guide for selecting beneficial monarch host plants.

## Supporting information

S1 Appendix Literature review supplement. Includes supplemental methods (including PRISMA resources), supplemental results (included studies and host classes for plant species), and PRISMA checklist. (A Fig and A and B Tables).
(DOCX)

S2 Appendix Meta-analysis supplement. Includes hypotheses, supplemental methods (including PRISMA resources, and phylogenetic analysis), and supplemental results (included studies, mean character values for plant species, model details, and phylogenetic analysis results). (A and B Figs and A-I Tables).
(DOCX)

S3 Appendix Experimental supplement. Includes supplemental methods and model details. (A-F Tables).
(DOCX)

S1 File PRISMA checklist. (DOCX)
