# Supplementary material for: Host plant specificity of the monarch butterfly Danaus plexippus: A systematic review and meta-analysis
Source: PLoS One. 2022 Jun 14;17(6):e0269701. doi: 10.1371/journal.pone.0269701 (PMC9197062; doi:10.1371/journal.pone.0269701)
Supplement: S1 Appendix — Includes supplemental methods (including PRISMA resources), supplemental results (included studies and host classes for plant species), and PRISMA checklist. (A Fig and A and B Tables). (DOCX) [file pone.0269701.s001.docx]

**S1 Appendix: Literature review supplement**

**Supplemental methods**

**A Fig:** Flow chart (modified from PRISMA flow diagrams) summarizing search methods for the literature review.

Google Scholar was used because it covers a broad range of publication types, has been estimated to contain at least 100 million scholarly documents [1], and because Google Scholar easily integrates with the software used to automate and conduct searches: Publish or Perish [2].

Searches for palatability data were conducted on April 22, 2021. The search terms used followed the format: {GBIF species name} Danaus plexippus. Peer reviewed publications, conference papers, and academic theses were included in analyses. If this search returned no results, “Research Grade” iNaturalist observations of the plant were manually screened for co-observations with monarch larvae. See A Fig in S1 Appendix for the number of sources at each step of the PRISMA process. Studies meeting the following criteria were excluded from use in this study:

- A study is irrelevant to or contains no data on associations between the plant of interest and monarch butterflies.
- A study is a review presenting or analyzing previously published data or information.
- A study is poorly controlled (e.g., larval survival and/or performance may be affected by non-plant related factors including environmental conditions, predation, or parasitism).

All statistical analyses were conducted using RStudio 2021.09.0+351 "Ghost Orchid" Release for macOS [3] running R version 4.1.1 [4]. R scripts are available upon request. The following R packages were used: tidyverse [5] and ggpubr [6].

**Supplemental results**

Sources included in this study’s literature review with notes are presented in A Table in S1 Appendix.

**A Table:** Sources of palatability data included in this study

| **Authors** | **Year** | **Citation** | **Notes** |
| --- | --- | --- | --- |
| Ackery and Vane-Wright | 1984 | [7] |  |
| Adams et al. | 2021 | [8] | Uses a synonymous species name |
| Agrawal and Hastings | 2019 | [9] |  |
| Agrawal et al. | 2009 | [10] |  |
| Agrawal et al. | 2014 | [11] |  |
| Alonso-Mejía and Brower | 1994 | [12] |  |
| Betz | 1989 | [13] | Conference paper |
| Betz et al. | 1997 | [14] | Conference paper |
| Borkin | 1991 | [15] |  |
| Brock | 2009 | [16] |  |
| Brower | 1977 | [17] | Uses a synonymous species name |
| Brower et al | 1984 | [18] |  |
| Brower et al. | 1967 | [19] |  |
| Brower et al. | 1975 | [20] |  |
| Brower et al. | 1982 | [21] |  |
| Burns | 1983 | [22] | Uses a synonymous species name |
| Casagrande and Dacey | 2014 | [23] |  |
| de La Pava and Sepúlveda | 2012 | [24] |  |
| DiTommaso and Losey | 2003 | [25] |  |
| Dixon et al. | 1977 | [26] |  |
| Edgar et al. | 1976 | [27] | Uses a synonymous species name |
| Elliott et al. | 2009 | [28] | Uses a synonym and accepted name interchangeably |
| Erickson | 1973 | [29] |  |
| Gil-T | 2006 | [30] |  |
| Grodsky et al. | 2020 | [31] |  |
| Groeneveld et al. | 1990 | [32] | Uses a synonymous species name |
| Harinath and Meera Bai | 2014 | [33] |  |
| Higgins | 1970 | [34] | Conflicts with other reports |
| Hoang et al. | 2017 | [35] |  |
| Johnson et al. | 2014 | [36] |  |
| Jones et al. | 2019 | [37] |  |
| Kimball | 1965 | [38] | Conflicts with other reports |
| Koch et al. | 1977 | [39] | Conflicts with other reports |
| Ladner and Altizer | 2005 | [40] |  |
| Lynch and Martin | 1987 | [41] |  |
| Lynch and Martin | 1991 | [42] |  |
| Malcolm and Brower | 1989 | [43] |  |
| Malcolm and Zalucki | 1996 | [44] |  |
| Martin and Lynch | 1988 | [45] |  |
| Matiella | 2009 | [46] |  |
| Mattila and Otis | 2003 | [47] |  |
| McKay et al. | 2016 | [48] |  |
| Minno | 1997 | [49] | Conflicts with other reports |
| Pegram and Melkonoff | 2020 | [50] |  |
| Pellissier et al. | 2016 | [51] |  |
| Pocius et al. | 2017 | [52] |  |
| Rasmann et al. | 2009 | [53] |  |
| Riley | 1991 | [54] |  |
| Roels | 2011 | [55] | Academic thesis |
| Schroeder et al. | 2020 | [56] |  |
| Stenoien et al. | 2019 | [57] |  |
| Tietz | 1972 | [58] | Conflicts with other reports |
| Urquhart | 1960 | [59] |  |
| Vickerman and de Boer | 2002 | [60] |  |
| Yeargan and Allard | 2005 | [61] |  |

A full list classifying all plant species claimed to have an association with monarch butterflies with notes is presented in B Table in S1 Appendix. Host classes are defined in Table 1 (main text).

**B Table:** Host classes of plant species with notes.

| **Plant Species** | **Class** | **Disputed Sources** | **Explanations and Notes** |
| --- | --- | --- | --- |
| *Achillea millefolium* | N [62] |  | Larvae do not attempt to eat. |
| *Ageratina altissima* | N [62] |  | Larvae do not attempt to eat. |
| *Apocynum androsaemifolium* | N [15] | [38,58,63] | Larvae do not attempt to eat. |
| *Apocynum cannabinum* | N [15,62] | [38] | Larvae do not attempt to eat or eat very little. |
| *Aquilegia canadensis* | N [62] |  | Larvae do not attempt to eat. |
| *Araujia sericifera* | L3 [28] |  | Citation documents claim. This plant was experimentally tested in this study. |
| *Asclepias albicans* | U | [64] |  |
| *Asclepias amplexicaulis* | H1 [42,54] |  | Many observations of different instar larvae on this plant. |
| *Asclepias angustifolia* | H3 [50] |  | Survival over 50% |
| *Asclepias arenaria* | L3 [51] | [65] | Larvae begin feeding, but the absence of data/observations suggests it is not a high performance host. |
| *Asclepias asperula* | H2 [43,45,46] |  | Larvae reared in multiple studies. |
| *Asclepias barjoniifolia* | H2 [37] |  | Larvae reared for a study on this plant. |
| *Asclepias boliviensis* | L3 [51,53] |  | Larvae begin feeding, but the absence of data/observations suggests it is not a high performance host. |
| *Asclepias brachystephana* | L3 [51] |  | Larvae begin feeding, but the absence of data/observations suggests it is not a high performance host. |
| *Asclepias californica* | H2 [18] |  | Larvae reared for a study on this plant. |
| *Asclepias candida* | L3 [51] |  | Larvae begin feeding, but the absence of data/observations suggests it is not a high performance host. |
| *Asclepias cinerea* | U | [66] |  |
| *Asclepias connivens* | U | [66] |  |
| *Asclepias cordifolia* | H2 [43] |  | Larvae reared in multiple studies. |
| *Asclepias cryptoceras* | L3 [51] |  | Larvae begin feeding, but the absence of data/observations suggests it is not a high performance host. |
| *Asclepias curassavica* | H3 [29,57] | [66] | Survival over 50% |
| *Asclepias curtissii* | U | [63] |  |
| *Asclepias engelmanniana* | L3 [51] | [65] | Larvae begin feeding, but the absence of data/observations suggests it is not a high performance host. |
| *Asclepias eriocarpa* | H2 [21] |  | Larvae reared for a study on this plant. |
| *Asclepias erosa* | H2 [43] |  | Larvae reared for a study on this plant. |
| *Asclepias exaltata* | H3 [52] |  | Survival over 50% |
| *Asclepias fascicularis* | H3 [26,40] |  | Survival over 50% |
| *Asclepias glaucescens* | L3 [51,67] |  | Larvae begin feeding, but the absence of data/observations suggests it is not a high performance host. |
| *Asclepias hallii* | L3 [11,51] |  | Larvae begin feeding, but the absence of data/observations suggests it is not a high performance host. |
| *Asclepias hirtella* | L3 [52] | [63,65] | Fewer than 50% of larvae reared on this plant survived to adulthood. |
| *Asclepias humistrata* | H2 [12,20,43] |  | Larvae reared in multiple studies |
| *Asclepias incarnata* | H3 [29,40,48,56,57] |  | Survival over 50% |
| *Asclepias labriformis* | L3 [9] |  | Larvae begin feeding, but the absence of data/observations suggests it is not a high performance host. |
| *Asclepias lanceolata* | H1 [59] |  | Many observations of different instar larvae on this plant. |
| *Asclepias lanuginosa* | L1 [14] |  | Isolated observations of larvae. |
| *Asclepias latifolia* | H2 [35] |  | Larvae reared to adult for a study. |
| *Asclepias lemmonii* | L3 [51] |  | Larvae begin feeding, but the absence of data/observations suggests it is not a high performance host. |
| *Asclepias linaria* | L3 [50] |  | Fewer than 50% of larvae reared on this plant survived to adulthood. |
| *Asclepias longifolia* | H1 [54] |  | Many observations of different instar larvae on this plant. |
| *Asclepias meadii* | L1 [13,14,55] | [64] | Isolated observations of larvae. |
| *Asclepias michauxii* | U | [66] |  |
| *Asclepias nivea* | H2 [68] |  | Larvae reared to adult for a study. |
| *Asclepias nyctaginifolia* | L1 [31] | [64] | Isolated observations of larvae. |
| *Asclepias obovata* | L3 [11,51] | [63,66] | Larvae begin feeding, but the absence of data/observations suggests it is not a high performance host. |
| *Asclepias oenotheroides* | L3 [51] | [63,69] | Larvae begin feeding, but the absence of data/observations suggests it is not a high performance host. |
| *Asclepias otarioides* | L3 [51] |  | Larvae begin feeding, but the absence of data/observations suggests it is not a high performance host. |
| *Asclepias ovalifolia* | L3 [51] | [65] | Larvae begin feeding, but the absence of data/observations suggests it is not a high performance host. |
| *Asclepias pedicellata* | U | [66] |  |
| *Asclepias perennis* | H2 [37] |  | Survival over 50% |
| *Asclepias pumila* | L3 [51,53] | [65] | Larvae begin feeding, but the absence of data/observations suggests it is not a high performance host. |
| *Asclepias purpurascens* | H1 [16] |  | Many observations of different instar larvae on this plant. |
| *Asclepias quadrifolia* | L3 [51] | [65,66] | Larvae begin feeding, but the absence of data/observations suggests it is not a high performance host. |
| *Asclepias rubra* | L3 [51] | [66] | Larvae begin feeding, but the absence of data/observations suggests it is not a high performance host. |
| *Asclepias solanoana* | L3 [51] |  | Larvae begin feeding, but the absence of data/observations suggests it is not a high performance host. |
| *Asclepias speciosa* | H3 [40,52,70] |  | Survival over 50% |
| *Asclepias stenophylla* | L3 [9] | [65] | Larvae begin feeding, but the absence of data/observations suggests it is not a high performance host. |
| *Asclepias subulata* | L3 [51] | [63,64,69] | Larvae begin feeding, but the absence of data/observations suggests it is not a high performance host. |
| *Asclepias subverticillata* | L3 [51,53] | [63,64] | Larvae begin feeding, but the absence of data/observations suggests it is not a high performance host. |
| *Asclepias sullivantii* | L3 [52] | [64,65] | Fewer than 50% of larvae reared on this plant survived to adulthood. |
| *Asclepias syriaca* | H3 [29,36,40,44,52,61,71] |  | Survival over 50% |
| *Asclepias texana* | L3 [51,53] |  | Larvae begin feeding, but the absence of data/observations suggests it is not a high performance host. |
| *Asclepias tomentosa* | U | [58,63,64,66] |  |
| *Asclepias tuberosa* | H3 [29,52] |  | Survival over 50% |
| *Asclepias variegata* | L3 [51] | [63–66,69] | Larvae begin feeding, but the absence of data/observations suggests it is not a high performance host. |
| *Asclepias verticillata* | H3 [52] |  | Survival over 50% |
| *Asclepias vestita* | H2 [43] |  | Larvae reared for a study on this plant |
| *Asclepias viridiflora* | H1 [42,51] |  | Many observations of different instar larvae on this plant. |
| *Asclepias viridis* | H1 [41,42] |  | Many observations of different instar larvae on this plant. |
| *Baptisia alba* | N [62] |  | Larvae do not attempt to eat. |
| *Brassica oleracea var. capitata* | N [19] |  | Brower et al. 1967 [19] selectively bred larvae to feed and survive on this plant, but larvae did not feed without intervention. |
| *Calotropis gigantea* | H2 [26] |  | Larvae reared to adulthood on this plant. |
| *Calotropis procera* | H3 [24] |  | Survival over 50% |
| *Catharanthus roseus* | L2 [62] |  | Larvae begin feeding but die before reaching adulthood. |
| *Ceropegia grandiflora* | U | [39] |  |
| *Ceropegia mixta* | N [62] | [39,63] | Larvae do not attempt to eat. |
| *Chamaecrista fasciculata* | N [62] |  | Larvae do not attempt to eat. |
| *Coreopsis tinctoria* | N [62] |  | Larvae do not attempt to eat. |
| *Cynanchum acutum* | H1 [30] |  | Many observations of different instar larvae on this plant. |
| *Cynanchum laeve* | H3 [52,61] |  | Survival over 50% |
| *Cynanchum pulchellum* | L1 [33] | [63] | Adults occasionally oviposit on this plant, but the absence of data/observations suggests it is not a high performance host. |
| *Dalea purpurea* | N [62] |  | Larvae do not attempt to eat. |
| *Datura innoxia* | L2 [62] |  | Larvae begin feeding but die before reaching adulthood. |
| *Dictyanthus reticulatus* | L3 [62] | [63] | Larvae develop very slowly to at least the third instar and suffer high mortality. |
| *Digitalis purpurea* | L2 [62] |  | Larvae begin feeding but die before reaching adulthood. |
| *Echinacea paradoxa* | N [62] |  | Larvae do not attempt to eat. |
| *Euphorbia mauritanica* | U | [34,63] | Ackery and Vane-Wright 1984 [7] list this plant as a “very doubtful” host; Dixon et al. 1977 [26] suggests this inclusion may be the result of a linguistic error. |
| *Funastrum clausum* | U | [49,63] |  |
| *Glandularia canadensis* | N [62] |  | Larvae do not attempt to eat. |
| *Gomphocarpus cancellatus* | H1 [27] |  | Many observations of different instar larvae on this plant. |
| *Gomphocarpus fruticosus* | H2 [26,32] |  | Larvae reared on this plant for multiple studies. |
| *Gomphocarpus physocarpus* | H2 [8] |  | Larvae reared on this plant for a study. Despite using a synonymous species name, abundant co-observations on iNaturalist support this classification. |
| *Gonolobus rostratus* | H2 [72] |  | Larvae reared on this plant for a study. |
| *Gonolobus suberosus* | L3 [7] | [66] | This plant was experimentally tested in this study. |
| *Gossypium arboretum* | U | [34,63] | Ackery and Vane-Wright 1984 [7] list this plant as a “very doubtful” host; Dixon et al. 1977 [26] suggests this inclusion may be the result of a linguistic error. |
| *Hibiscus trionum* | N [62] |  | Larvae do not attempt to eat. |
| *Hoya carnosa* | N [26] |  | Larvae do not attempt to eat. |
| *Ipomoea batatas* | N [26] | [38] | Larvae do not attempt to eat. |
| *Liatris pycnostachya* | N [62] |  | Larvae do not attempt to eat. |
| *Lobelia siphilitica* | N [62] |  | Larvae do not attempt to eat. |
| *Lycopersicon esculentum* | L2 [62] |  | Larvae begin feeding but die before reaching adulthood. |
| *Matelea carolinensis* | U | [66] |  |
| *Matelea decipiens* | U | [66] |  |
| *Matelea flavidula* | U | [66] |  |
| *Monarda fistulosa* | N [62] |  | Larvae do not attempt to eat. |
| *Nerium oleander* | N [26] |  | Larvae do not attempt to eat. |
| *Nicandra physalodes* | N [62] |  | Larvae do not attempt to eat. |
| *Oenothera biennis* | N [67] |  | Larvae do not attempt to eat. |
| *Orthosia scoparia* | U | [66] |  |
| *Oxypetalum alpinum* | U | [7,63] |  |
| *Oxypetalum coeruleum* | L2 [62] | [63] | Larvae begin feeding but die before reaching adulthood. |
| *Pachycarpus grandifloras* | U | [17,63] |  |
| *Penstemon digitalis* | N [62] |  | Larvae do not attempt to eat. |
| *Pergularia daemia* | U |  | Brower et al. 1975 [20] imply this is not a host plant. |
| *Petroselinum crispum* | N [62] |  | Larvae do not attempt to eat. |
| *Phaseolus lunatus* | N [67] |  | Larvae do not attempt to eat. |
| *Phlox pilosa* | N [62] |  | Larvae do not attempt to eat. |
| *Phytolacca americana* | N [62] |  | Larvae do not attempt to eat. |
| *Plumbago zeylanica* | N [62] |  | Larvae do not attempt to eat. |
| *Rudbeckia hirta* | N [62] |  | Larvae do not attempt to eat. |
| *Salvia azurea* | N [62] |  | Larvae do not attempt to eat. |
| *Seutera angustifolia* | L1 [22] | [63,66] | Adults occasionally oviposit on this plant, but the absence of data/observations suggests it is not a high performance host. |
| *Solanum dulcamara* | N | [59] | This plant was experimentally tested in this study. |
| *Solidago canadensis* | N [62] |  | Larvae do not attempt to eat. |
| *Stephanotis floribunda* | N [26] | [63] | Larvae do not attempt to eat. |
| *Vigna unguiculata* | L2 [62] |  | Larvae begin feeding but die before reaching adulthood. |
| *Vincetoxicum nigrum* | L2 [23,25] |  | Larvae begin feeding but die before reaching adulthood. |
| *Vincetoxicum rossicum* | L2 [23,25,47] |  | Larvae begin feeding but die before reaching adulthood. |

**References**

1. Khabsa M, Giles CL. The Number of Scholarly Documents on the Public Web. PLOS ONE. 2014;9: e93949. doi:10.1371/journal.pone.0093949

2. Harzing AW. Publish or Perish. 2007. Available: https://harzing.com/resources/publish-or-perish

3. RStudio Team. RStudio: Integrated Development Environment for R. Boston, MA: RStudio, PBC; 2021. Available: http://www.rstudio.com/

4. R Core Team. R: A Language and Environment for Statistical Computing. Vienna, Austria: R Foundation for Statistical Computing; 2021. Available: https://www.R-project.org/

5. Wickham H, Averick M, Bryan J, Chang W, McGowan LD, François R, et al. Welcome to the Tidyverse. Journal of Open Source Software. 2019;4: 1686. doi:10.21105/joss.01686

6. Kassambara A. ggpubr: “ggplot2” Based Publication Ready Plots. 2020. Available: https://CRAN.R-project.org/package=ggpubr

7. Ackery P, Vane-Wright R. Milkweed Butterflies, Their Cladistics and Biology: Being an Account of the Natural History of the Danainae, a Subfamily of the Lepidoptera, Nymphalidae. London: British Museum of Natural History; 1984.

8. Adams KL, Aljohani A, Chavez J, de Roode JC. Effects of cardenolides of milkweed plants on immunity of the monarch butterfly. Arthropod-Plant Interactions. 2021;15: 249–252. doi:10.1007/s11829-021-09812-w

9. Agrawal AA, Hastings AP. Trade-offs constrain the evolution of an inducible defense within but not between plant species. Ecology. 2019;100: e02857. doi:https://doi.org/10.1002/ecy.2857

10. Agrawal AA, Fishbein M, Jetter R, Salminen J-P, Goldstein JB, Freitag AE, et al. Phylogenetlc Ecology of Leaf Surface Traits in the Milkweeds (Asclepias spp.): Chemistry, Ecophysiology, and Insect Behavior. The New Phytologist. 2009;183: 848–867.

11. Agrawal AA, Hastings AP, Patrick ET, Knight AC. Specificity of Herbivore-Induced Hormonal Signaling and Defensive Traits in Five Closely Related Milkweeds (Asclepias spp.). J Chem Ecol. 2014;40: 717–729. doi:10.1007/s10886-014-0449-6

12. Alonso-Mejía A, Brower LP. From model to mimic: Age-dependent unpalatability in monarch butterflies. Experientia. 1994;50: 176–181. doi:10.1007/BF01984960

13. Betz RF. Ecology of Mead’s Milkweed (Asclepias meadii Torrey). Proceedings of the North American Prairie Conference. Lincoln, Nebraska; 1989. p. 6. Available: https://core.ac.uk/download/pdf/188059322.pdf

14. Betz RF, Rommel WR, Dichtl JJ. Insect herbivores of 12 milkweed (Asclepias) species. In: Warwick C, editor. Proceedings of the Fifteenth North American Prairie Conference. Bend, OR: Natural Areas Association; 2000. pp. 7–19.

15. Borkin SS. Rejection of Apocynum Androsaemifolium and A. sibiricum (Apocynaceae) as Food Plants by Larvae of Danaus plexippus: Refutation of Early Accounts. In: Malcolm SB, Zalucki MP, editors. Biology and Conservation of the Monarch Butterfly. Los Angeles, Calif: Natural History Museum of Los Angeles County; 1991. pp. 107–125.

16. Brock TD. Ecology and Conservation of Purple Milkweed. Ecological Rest. 2009;27: 269–277. doi:10.3368/er.27.3.269

17. Brower LP. Monarch Migration. American Museum of Natural History. 1977;86: 40–53.

18. Brower LP, Seiber JN, Nelson CJ, Lynch SP, Hoggard MP, Cohen JA. Plant-determined variation in cardenolide content and thin-layer chromatography profiles of monarch butterflies, Danaus plexippus reared on milkweed plants in California: 3. Asclepias californica. J Chem Ecol. 1984;10: 1823–1857. doi:10.1007/BF00987364

19. Brower LP, Brower JVZ, Corvino JM. Plant poisons in a terrestrial food chain. PNAS. 1967;57: 893–898. doi:10.1073/pnas.57.4.893

20. Brower LP, Edmunds M, Moffitt CM. Cardenolide content and palatability of a population of Danaus chrysippus butterflies from West Africa. Journal of Entomology Series A, General Entomology. 1975;49: 183–196. doi:10.1111/j.1365-3032.1975.tb00084.x

21. Brower LP, Seiber JN, Nelson CJ, Lynch SP, Tuskes PM. Plant-determined variation in the cardenolide content, thin-layer chromatography profiles, and emetic potency of monarch butterflies, Danaus plexippus reared on the milkweed, Asclepias eriocarpa in California. J Chem Ecol. 1982;8: 579–633. doi:10.1007/BF00989631

22. Burns J. Queen of the Carolinas (Lepidoptera: Nymphalidae: Danainae: Danaus gilippus). Proceedings of the Entomological Society of Washington. 1983;85: 388–396.

23. Casagrande RA, Dacey JE. Monarch Butterfly Oviposition on Swallow-Worts (Vincetoxicum spp.). Environmental Entomology. 2007;36: 631–636. doi:10.1603/0046-225X(2007)36[631:MBOOSV]2.0.CO;2

24. de La Pava N, Sepúlveda PA. Aspectos del desarrollo de Danaus plexippus (Lepidoptera: Nymphalidae) sobre Calotropis procera (Apocynaceae) bajo condiciones de laboratorio. Boletín Científico Centro de Museos Museo de Historia Natural. 2012;16: 266–272.

25. DiTommaso A, Losey JE. Oviposition preference and larval performance of monarch butterflies (Danaus plexippus) on two invasive swallow-wort species. Entomologia Experimentalis et Applicata. 2003;108: 205–209. doi:10.1046/j.1570-7458.2003.00089.x

26. Dixon CA, Erickson JM, Kellett DN, Rothschild M. Some adaptations between Danaus plexippus and its food plant, with notes on Danaus chrysippus and Euploea core (Insecta: Lepidoptera). Journal of Zoology. 1978;185: 437–467. doi:10.1111/j.1469-7998.1978.tb03344.x

27. Edgar JA, Cockrum PA, Frahn JL. Pyrrolizidine alkaloids in Danaus plexippus L. and Danaus chrysippus L. Experientia. 1976;32: 1535–1537. doi:10.1007/BF01924437

28. Elliott MS, Massey B, Cui X, Hiebert E, Charudattan R, Waipara N, et al. Supplemental host range of Araujia mosaic virus, a potential biological control agent of moth plant in New Zealand. Austral Plant Pathol. 2009;38: 603. doi:10.1071/AP09046

29. Erickson JM. The Utilization of Various Asclepias Species by Larvae of the Monarch Butterfly, Danaus Plexippus. Psyche: A Journal of Entomology. 1973;80: 230–244. doi:10.1155/1973/28693

30. Gil-T F. A new hostplant for Danaus plexippus (Linnaeus, 1758) in Europe. A study of cryptic preimaginal polymorphism within Danaus chrysippus (Linnaeus, 1758) in southern Spain (Andalusia) (Lepidoptera, Nymphalidae, Danainae). Atalanta. 2006;37: 143–149.

31. Grodsky SM, Saul-Gershenz LS, Moore-O’Leary KA, Whitney JP, Hernandez RR. Hare don’t care! Consumption of a rare, desert milkweed containing phytochemicals by the black-tailed jackrabbit. Journal of Arid Environments. 2020;174: 103991. doi:10.1016/j.jaridenv.2019.103991

32. Groeneveld HW, Steijl H, Van Den Berg B, Elings JC. Rapid, quantitative HPLC analysis of Asclepias fruticosa L. and Danaus plexippus L. cardenolides. J Chem Ecol. 1990;16: 3373–3382. doi:10.1007/BF00982104

33. Harinath P, Meera Bai G, Venketa Raman S. Diversity of Butterflies - Strategies adopted for its conservation at Yogi Vemana University campus, Kadapa, A.P., India. Discovery. 2014;11: 34–51.

34. Higgins LG, Riley ND. A field guide to the butterflies of Britain and Europe. [1st American ed.]. Boston, MA: Houghton Mifflin; 1970. Available: http://hdl.handle.net/2027/umn.31951000090635g

35. Hoang K, Tao L, Hunter MD, de Roode JC. Host Diet Affects the Morphology of Monarch Butterfly Parasites. Journal of Parasitology. 2017;103: 228–236. doi:10.1645/16-142

36. Johnson H, Solensky MJ, Satterfield DA, Davis AK. Does Skipping a Meal Matter to a Butterfly’s Appearance? Effects of Larval Food Stress on Wing Morphology and Color in Monarch Butterflies. Smagghe G, editor. PLoS ONE. 2014;9: e93492. doi:10.1371/journal.pone.0093492

37. Jones PL, Petschenka G, Flacht L, Agrawal AA. Cardenolide Intake, Sequestration, and Excretion by the Monarch Butterfly along Gradients of Plant Toxicity and Larval Ontogeny. J Chem Ecol. 2019;45: 264–277. doi:10.1007/s10886-019-01055-7

38. Kimball CP. The Lepidoptera of Florda: An Annotated Checklist. Gainesville, Florida: Florida Department of Agriculture; 1965.

39. Koch LE, Dell B, Keighery GJ. The Wanderer Butterfly at Bunbury and Other Parts of the South-West, and a New Food Plant. The Western Australian Naturalist. 1977;13: 183–184.

40. Ladner DT, Altizer S. Oviposition preference and larval performance of North American monarch butterflies on four Asclepias species. Entomologia Experimentalis et Applicata. 2005;116: 9–20. doi:https://doi.org/10.1111/j.1570-7458.2005.00308.x

41. Lynch SP, Martin RA. Cardenolide Content and Thin-Layer Chromatography Profiles of Monarch Butterflies, Danaus plexippus L., and their Larval Host-Plant Milkweed, Asclepias viridis Walt., in Northwestern Louisiana. Journal of Chemical Ecology. 1987;13: 24.

42. Lynch SP, Martin RA. Milkweed Host Plant Utilization and Cardenolide Sequestration by Monarch Butterflies in Louisiana and Texas. In: Malcolm SB, Zalucki MP, editors. Biology and Conservation of the Monarch Butterfly. Los Angeles, Calif: Natural History Museum of Los Angeles County; 1991. pp. 107–125.

43. Malcolm SB, Brower LP. Evolutionary and Ecological Implications of Cardenolide Sequestration in the Monarch Butterfly. Experientia. 1989;45: 284–295. doi:10.1007/BF01951814

44. Malcolm SB, Zalucki MP. Milkweed latex and cardenolide induction may resolve the lethal plant defence paradox. In: Städler E, Rowell-Rahier M, Bauer R, editors. Proceedings of the 9th International Symposium on Insect-Plant Relationships. Dordrecht: Springer Netherlands; 1996. pp. 193–196. doi:10.1007/978-94-009-1720-0_44

45. Martin RA, Lynch SP. Cardenolide Content and Thin-Layer Chromatography Profiles of Monarch Butterflies, Danaus plexippus L., and Their Larval Host-Plant Milkweed, Asclepias asperula subsp. capricornu (Woods.) Woods., in North Central Texas. Journal of Chemical Ecology. 1988;14: 24.

46. Matiella TJ. The effects of carbon dioxide on three species of milkweed (Asclepiadaceae) and monarch butterfly (Danaus plexippus) larva feeding preference. M.S., The University of Texas at San Antonio. 2009. Available: https://www.proquest.com/docview/305158906/abstract/37CA2463ADC640B3PQ/1

47. Mattila HR, Otis GW. A comparison of the host preference of monarch butterflies (Danaus plexippus) for milkweed (Asclepias syriaca) over dog-strangler vine (Vincetoxicum rossicum). Entomologia Experimentalis et Applicata. 2003;107: 193–199. doi:10.1046/j.1570-7458.2003.00049.x

48. McKay AF, Ezenwa VO, Altizer S. Consequences of Food Restriction for Immune Defense, Parasite Infection, and Fitness in Monarch Butterflies. Physiological and Biochemical Zoology. 2016;89: 389–401. doi:10.1086/687989

49. Minno MC. Butterflies of Florida’s Wetlands Part 1: Swallowtails, Whites, and Milkweed Butterflies. Aquatics. 199719: 14–18.

50. Pegram KV, Melkonoff NA. Assessing preference and survival of Danaus plexippus on two western species of Asclepias. J Insect Conserv. 2020;24: 287–295. doi:10.1007/s10841-019-00197-z

51. Pellissier L, Litsios G, Fishbein M, Salamin N, Agrawal AA, Rasmann S. Different rates of defense evolution and niche preferences in clonal and nonclonal milkweeds (Asclepias spp.). New Phytologist. 2016;209: 1230–1239. doi:10.1111/nph.13649

52. Pocius VM, Debinski DM, Pleasants JM, Bidne KG, Hellmich RL, Brower LP. Milkweed Matters: Monarch Butterfly (Lepidoptera: Nymphalidae) Survival and Development on Nine Midwestern Milkweed Species. Environ Entomol. 2017;46: 1098–1105. doi:10.1093/ee/nvx137

53. Rasmann S, Agrawal AA, Cook SC, Erwin AC. Cardenolides, induced responses, and interactions between above- and belowground herbivores of milkweed (Asclepias spp.). Ecology. 2009;90: 2393–2404. doi:10.1890/08-1895.1

54. Riley TJ. Spring Migration and Ovipositions of the Monarch Butterfly in Louisiana. In: Malcolm SB, Zalucki MP, editors. Biology and Conservation of the Monarch Butterfly. Los Angeles, Calif: Natural History Museum of Los Angeles County; 1991. pp. 107–125.

55. Roels SM. Not Easy Being Mead’s: Comparative Herbivory on Three Milkweeds, Including Threatened Mead’s Milkweed (Asclepias meadii), and Seedling Ecology of Mead’s Milkweed. Thesis, University of Kansas. 2011. Available: https://kuscholarworks.ku.edu/handle/1808/8183

56. Schroeder H, Majewska A, Altizer S. Monarch butterflies reared under autumn-like conditions have more efficient flight and lower post-flight metabolism. Ecological Entomology. 2020;45: 562–572. doi:10.1111/een.12828

57. Stenoien CM, Meyer RA, Nail KR, Zalucki MP, Oberhauser KS. Does chemistry make a difference? Milkweed butterfly sequestered cardenolides as a defense against parasitoid wasps. Arthropod-Plant Interactions. 2019;13: 835–852. doi:10.1007/s11829-019-09719-7

58. Tietz HM. An Index to the Described Life Histories, Early Stages and Hosts of the Macrolepidoptera of the Continental United States and Canada. Sarasota, Florida: The Allyn Museum of Entomology; 1972.

59. Urquhart FA. The Monarch Butterfly. Toronto, Canada: University of Toronto Press; 1960.

60. Vickerman DB, de Boer G. Maintenance of narrow diet breadth in the monarch butterfly caterpillar: response to various plant species and chemicals. Entomologia Experimentalis et Applicata. 2002;104: 255–269. doi:10.1046/j.1570-7458.2002.01012.x

61. Yeargan KV, Allard CM. Comparison of Common Milkweed and Honeyvine Milkweed (Asclepiadaceae) as Host Plants for Monarch Larvae (Lepidoptera: Nymphalidae). Journal of the Kansas Entomological Society. 2005;78: 247–251. doi:10.2317/0407.40.1

62. Vickerman DB, Boer G de. Maintenance of narrow diet breadth in the monarch butterfly caterpillar: response to various plant species and chemicals. Entomologia Experimentalis et Applicata. 2002;104: 255–269. doi:10.1046/j.1570-7458.2002.01012.x

63. Robinson GS, Ackery P, Kitching IJ, Beccaloni GW, Hernández LM. HOSTS - A Database of the World’s Lepidopteran Hostplants. London: Natural History Museum; 2010. Available: http://www.nhm.ac.uk/hosts

64. Borders B, Lee-Mäder E. Milkweeds: A Conservation Practitioner’s Guide. Portland, OR: The Xerces Society for Invertebrate Conservation; 2014.

65. Milkweed Species Beneficial to the Monarch Butterfly. In: United States Forest Service [Internet]. [cited 17 Sep 2021]. Available: https://www.fs.fed.us/wildflowers/pollinators/Monarch_Butterfly/habitat/milkweed_list.shtml

66. Conservation Cover (Monarch Habitat). Natural Resources Conservation Service; 2015.

67. Agrawal AA, Salminen J-P, Fishbein M. Phylogenetic Trends in Phenolic Metabolism of Milkweeds (asclepias): Evidence for Escalation. Evolution. 2009;63: 663–673. doi:10.1111/j.1558-5646.2008.00573.x

68. Tahsler BD. The distribution of cardenolides in Asclepias curassavica and A. nivea and its effect on the uptake of cardenolide dynamics of natural monarch populations. B.A., Amherst College. 1975.

69. Plant Milkweed for Monarchs. Monarch Joint Venture; Available: https://monarchjointventure.org/images/uploads/documents/MilkweedFactSheetFINAL.pdf

70. Freedman MG, Jason C, Ramírez SR, Strauss SY. Host plant adaptation during contemporary range expansion in the monarch butterfly. Evolution. 2020;74: 377–391. doi:10.1111/evo.13914

71. Fisher KE, Hellmich RL, Bradbury SP. Estimates of common milkweed (Asclepias syriaca) utilization by monarch larvae (Danaus plexippus) and the significance of larval movement. J Insect Conserv. 2020;24: 297–307. doi:10.1007/s10841-019-00213-2

72. Brower LP, McEvoy PB, Williamson KL, Flannery MA. Variation in cardiac glycoside content of monarch butterflies from natural populations in eastern North America. Science. 1972;177: 426–428. doi:10.1126/science.177.4047.426
