# Supplementary material for: Host plant specificity of the monarch butterfly Danaus plexippus: A systematic review and meta-analysis
Source: PLoS One. 2022 Jun 14;17(6):e0269701. doi: 10.1371/journal.pone.0269701 (PMC9197062; doi:10.1371/journal.pone.0269701)
Supplement: S2 Appendix — Includes hypotheses, supplemental methods (including PRISMA resources, and phylogenetic analysis), and supplemental results (included studies, mean character values for plant species, model details, and phylogenetic analysis results). (A and B Figs and A-I Tables). (DOCX) [file pone.0269701.s002.docx]

**S2 Appendix: Meta-analysis supplement**

A Table: Predictions and justifications of the meta-analysis

| **Property** | **Prediction** | **Justification** |
| --- | --- | --- |
| Trichome density | ↑ trichomes = ↓ likelihood of palatability | Trichomes act as barriers, slowing and/or preventing larvae from feeding [1–7] |
| Cardenolide concentration | ↑ concentration = ↓ likelihood of palatability | In high doses, cardenolides negatively affect larval fitness [8–12]. |
| Cardenolide polarity | ↓ polarity = ↓ likelihood of palatability | Cardenolides with lower polarity are better able to pass through the insect gut and are therefore more toxic [2,13,14]. |
| Cardenolide diversity | ↑ diversity = ↓ likelihood of palatability | The screening hypothesis states that plants produce a wide variety of secondary metabolites to reduce herbivory by decreasing herbivore performance through chemical interactions and additive effects [15–18]. |

**Supplemental methods**

**A Fig:** Flow charts (modified from PRISMA flow diagrams) summarizing meta-analysis search methods for data on A) cardenolides and B) trichome density.

Google Scholar was used because it covers a broad range of publication types, has been estimated to contain at least 100 million scholarly documents [19], and because Google Scholar easily integrates with the software used to automate and conduct searches: Publish or Perish [20].

**Cardenolides (Part A of A Fig in S2 Appendix)**

Searches for cardenolide data were conducted on April 22, 2021. Multiple search terms were used in an effort to collect data on non-cardenolide producing plants. For a given species, first the term “{GBIF species name} cardenolides” was used. If this did not return any results, the term “{GBIF species name} secondary metabolites” was searched. If this did not return any results, the term “{GBIF species name} extract” was used. Peer reviewed publications, conference papers, and academic theses were included in analyses. Studies meeting the following criteria were excluded from use in this study:

- A study is irrelevant to or contains no data on cardenolides in the plant of interest.
- A study does not specify which plant tissue was analyzed or a non-aerial tissue was analyzed.
- A study uses incompatible methods (e.g., non-polar extraction) or presents data in incompatible units (e.g., molarity).
- A study lists concentrations of individual cardenolide compounds, but not gross cardenolide concentrations.
- A study identifies individual cardenolide compounds but not concentrations, polarity, or diversity data.
- A study experimentally manipulates plant cardenolide content and includes no control values. Note that data from control plants were included in analysis when available.

**Trichome density (Part B of A Fig in S2 Appendix)**

Searches for cardenolide data were conducted on May 2, 2021. The search terms used followed the format: {GBIF species name} trichome density. See part B of A Fig in S2 Appendix for the number of sources at each step of the PRISMA process. Studies meeting the following criteria were excluded from use in this study:

- A study is irrelevant to or contains no data on trichome densities of the plant of interest.
- A study does not specify which surface(s)/tissue a trichome density is from.
- A study reports the density or number of only specific types of trichomes
- A study is a review presenting or analyzing previously published data or information.
- A study uses incompatible methods or presents data in incompatible units (e.g., density indices).
- A study experimentally manipulates plant trichome densities and includes no control values. Note that data from control plants were included in analysis when available.

**Statistical analysis**

All statistical analyses were conducted using RStudio 2021.09.0+351 "Ghost Orchid" Release for macOS [21] running R version 4.1.1 [22]. R scripts are available upon request. The following R packages were used: tidyverse [23], ggpubr [24], phytools [25], ape [26], cowplot [27], lme4 [28], emmeans [29], survival [30], survminer [31], coxme [32], caper [33], and stargazer [34].

Prior to conducting Wilcoxon rank sum tests, Shapiro-Wilk normality tests were conducted to test if the data were normally distributed, and F Tests were used to compare variances of the two samples.

We attempted to conduct a phylogenetic ANOVA (“phylANOVA” function from phytools [25]) to determine if cardenolide concentrations were significantly different between high and low performance hosts after genetic relatedness was accounted for.

To test the phylogenetic signals of performance status and mean cardenolide concentration, we calculated Fritz and Purvis’ D (binary variable; “phylo.d” from caper [33]) and Blomberg’s K (continuous variable; “phylosig” from phytools [25]) respectively. Tests were conducted using phylogeny data provided by Dr. Mark Fishbein and published in Fishbein et al. 2018 [35].

**Supplemental results**

Sources included in this study’s analysis of cardenolide properties are presented in B Table in S2 Appendix and sources included in analysis of trichome densities are presented in C Table in S2 Appendix.

**B Table:** Sources of cardenolide data included in this study; HPLC = high performance liquid chromatography, UPLC = ultra high performance liquid chromatography, GCMS = gas chromatography mass spectrometry, TNDP = 2',4,4'-tetranitrodiphenyl (spectrophotometric assay), DNBA = 3,5-dinitrobenzoic acid (Kedde reagent, spectrophotometric assay)

| **Authors** | **Year** | **Citation** | **Data type & method** | **Notes** |
| --- | --- | --- | --- | --- |
| Agrawal and Fishebein | 2016 | [36] | Concentration, HPLC |  |
| Agrawal and Hastings | 2019 | [37] | Concentration, HPLC |  |
| Agrawal et al. | 2009 | [38] | Concentration, HPLC |  |
| Agrawal et al. | 2014 | [39] | Concentration, HPLC |  |
| Agrawal et al. | 2015 | [40] | Concentration, HPLC |  |
| Agrawal et al. | 2021 | [41] | Concentration, HPLC |  |
| Ateya et al. | 2012 | [42] | Absence, HPLC | Ethanol extract of *H. trionum* notes no cardenolides |
| Bolsinger et al. | 1991 | [43] | Concentration, TNDP |  |
| Brower et al. | 1982 | [44] | Concentration, TNDP |  |
| Brower et al. | 1984 | [45] | Concentration, TNDP |  |
| Brower et al. | 1984 | [46] | Concentration, TNDP |  |
| Cohen and Brower | 1982 | [47] | Concentration, TNDP |  |
| Colvin et al. | 2013 | [48] | Concentration, DNBA |  |
| Dall’Acqua et al. | 2011 | [49] | Absence, HPLC | Methanol extract of *A. millefolium*; other glycosides identified but no cards |
| Decker et al. | 2019 | [50] | Concentration and diversity, HPLC |  |
| Decker et al. | 2019 | [51] | Concentration, diversity, and polarity, UPLC |  |
| DeLaMater et al. | 2021 | [52] | Concentration, Spectral prediction algorithm | Method validated by Couture et al. 2013 [53] |
| Dobler et al. | 1998 | [54] | Concentration, DNBA |  |
| El-Bakry et al. | 2011 | [55] | Concentration, DNBA |  |
| Erdman | 1983 | [56] | Concentration, TNDP |  |
| George | 2020 | [57] | Concentration, TNDP |  |
| Granica et al. | 2013 | [58] | Absence, UPLC | Methanol extract of *O. biennis* found no cardenolides |
| Hoang et al. | 2017 | [59] | Concentration, UPLC |  |
| Isman et al. | 1977 | [60] | Concentration, TNDP |  |
| Isman et al. | 1977 | [61] | Concentration, TNDP |  |
| Jones and Agrawal | 2019 | [62] | Concentration, HPLC |  |
| Kairuz et al. | 2020 | [63] | Concentration, HPLC |  |
| Lee et al. | 1994 | [64] | Absence, HPLC | Reports other steroid glycosides but no cardenolides |
| Lee et al. | 2016 | [65] | Absence, HPLC | Methanol extract of *I. batatas* found other steroid derived chemicals but no cardenolides |
| Lynch and Martin | 1987 | [66] | Concentration, TNDP |  |
| Malcolm | 1990 | [67] | Concentration, DNBA |  |
| Malcolm | 1991 | [2] | Concentration, TNDP | Data for *A. curtissi, A. exaltata, A. pediciliata, A. tomentosa, A. amplexicaulis, A. verticilliata* is unpublished; other numbers appear to be the middle of concentration ranges presented by Roeske et al. 1976 [68] and Seiber et al. 1983 [69] (*A. albicans, A. incarnata, A. subaphylla, A. subulata, A. tuberosa*) |
| Malcolm and Brower | 1986 | [70] | Concentration, TNDP |  |
| Malcolm and Brower | 1989 | [71] | Concentration, TNDP | Mostly a review, but include some unpublished data |
| Malcolm et al. | 1988 | [72] | Concentration, TNDP | Cite some other papers but also present unpublished data |
| Malcolm et al. | 1989 | [73] | Concentration, TNDP |  |
| Martel and Malcolm | 2004 | [74] | Concentration, HPLC |  |
| Martin and Lynch | 1988 | [75] | Concentration, TNDP |  |
| Martin et al. | 1992 | [76] | Concentration, DNBA |  |
| May | 1978 | [77] | Absence, GCMS | Academic thesis; ethanol extract of *A. altissima* notes no cardenolides |
| Moranz | 1996 | [78] | Concentration, TNDP |  |
| Nelson | 1988 | [79] | Concentration, TNDP | Cite some other papers but also present unpublished data |
| Nelson et al. | 1981 | [80] | Concentration, TNDP |  |
| Nishio et al. | 1983 | [81] | Concentration, TNDP |  |
| Oyeyele and Zalucki | 1990 | [82] | Concentration, DNBA |  |
| Palomino-Schätzlein et al. | 2017 | [83] | Absence, HPLC | Methanol extract of *A. sericifera* found other steroid compounds but no cardenolides |
| Park et al. | 2014 | [84] | Absence, GCMS | Methanol extract of *B. oleracea var capitata* finding no cardenolides |
| Rasmann and Agrawal | 2011 | [85] | Concentration, diversity, and polarity, HPLC |  |
| Rasmann et al. | 2009 | [13] | Concentration, HPLC |  |
| Ritland | 1991 | [86] | Concentration, TNDP |  |
| Roeske et al. | 1976 | [68] | Concentration, TNDP |  |
| Seiber et al | 1982 | [87] | Concentration, TNDP |  |
| Seiber et al. | 1985 | [88] | Concentration, HPLC | Conference paper |
| Seiber et al. | 1986 | [89] | Concentration, TNDP |  |
| Siddiqui et al. | 2011 | [90] | Absence, HPLC | Methanol extract of *V. rosea* found no cardenolides |
| Sternberg et al. | 2015 | [91] | Concentration, UPLC |  |
| Tahsler | 1975 | [92] | Concentration, TNDP |  |
| Tan et al. | 2019 | [93] | Concentration, UPLC |  |
| Tan et al. | 2019 | [94] | Concentration, UPLC |  |
| Tao et al. | 2014 | [11] | Concentration, UPLC |  |
| Van Hook and Zalucki | 1991 | [95] | Concentration, TNDP |  |
| Verma et al. | 2018 | [96] | Concentration, HPLC |  |
| Witte et al. | 1987 | [97] | Absence, GCMS |  |
| Yu et al. | 2017 | [98] | Absence, HPLC | Methanol extract of *N. physalodes* found withanolides but no cardenolides |
| Zalucki et al. | 1989 | [99] | Concentration, DNBA |  |
| Zalucki et al. | 1990 | [14] | Concentration, TNDP |  |
| Zalucki et al. | 2001 | [9] | Concentration, TNDP |  |
| Zehnder and Hunter | 2007 | [100] | Concentration, HPLC |  |
| Züst and Agrawal | 2016 | [101] | Concentration, HPLC |  |
| Züst et al. | 2018 | [102] | Concentration, HPLC |  |
| Züst et al. | 2019 | [103] | Concentration, HPLC |  |

**C Table:** Sources of trichome density data included in this study

| **Authors** | **Year** | **Citation** | **Notes** |
| --- | --- | --- | --- |
| Agrawal and Fishbein | 2008 | [104] |  |
| Agrawal and Fishbein | 2016 | [36] |  |
| Baker et al. | 2020 | [105] |  |
| Carvalho et al. | 2017 | [106] |  |
| Colvin | 2011 | [107] |  |
| Fishbein et al. | 2018 | [35] |  |
| Gómez-Nucamendi et al. | 2016 | [108] |  |
| Jones and Agrawal | 2019 | [62] |  |
| Maluf et al. | 2007 | [109] |  |
| Oghiakhe | 1995 | [110] |  |
| Oghiakhe et al. | 1992 | [111] |  |
| Rivas et al. | 2020 | [112] |  |
| Simmons et al. | 2003 | [113] |  |
| Verma and Chandra | 2014 | [114] |  |

Lists of mean cardenolide concentrations, mean cardenolide polarity/diversity, and mean trichome density for each species are listed in D, E, and F Tables (S2 Appendix) respectively. Wilcoxon tests found no significant differences between high and low performance in cardenolide polarity (Z = -1.0, p = 0.32), cardenolide diversity (Z = -0.68, p = 0.47), or trichome density (Z = -0.40, p = 0.69).

**D Table:** Mean cardenolide concentrations for plant species.

| **Plant Species** | **Mean Cardenolide Conc. (mg/g dry)** | **Source(s)** |
| --- | --- | --- |
| *Achillea millefolium* | 0 | [49] |
| *Ageratina altissima* | 0 | [77] |
| *Apocynum cannabinum* | 0.29 | [54] |
| *Araujia sericifera* | 0 | [83] |
| *Asclepias amplexicaulis* | 0.68 | [2,36,70,85] |
| *Asclepias angustifolia* | 0.65 | [13,38,85] |
| *Asclepias arenaria* | 1.63 | [85] |
| *Asclepias asperula* | 5.30 | [36–38,40,75,85,115] |
| *Asclepias barjoniifolia* | 4.09 | [13,85] |
| *Asclepias boliviensis* | 1.90 | [85] |
| *Asclepias brachystephana* | 2.81 | [85] |
| *Asclepias californica* | 2.10 | [36,38,40,45,61,85,87,115] |
| *Asclepias candida* | 4.00 | [85] |
| *Asclepias cordifolia* | 1.73 | [36,38,60,79,85,87] |
| *Asclepias cryptoceras* | 0.57 | [38,40,68,85,115] |
| *Asclepias curassavica* | 3.41 | [11,36,38,41,43,50,51,62,67,68,74,85,87,91–94,99,102,115] |
| *Asclepias curtissii* | 0.05 | [2] |
| *Asclepias engelmanniana* | 0.80 | [38,85] |
| *Asclepias eriocarpa* | 3.80 | [36,38,44,60,80,85,87] |
| *Asclepias erosa* | 3.21 | [38,61,79,85] |
| *Asclepias exaltata* | 0.74 | [2,36,38,85,115] |
| *Asclepias fascicularis* | 0.72 | [13,36–38,61,71,85] |
| *Asclepias glaucescens* | 1.62 | [38] |
| *Asclepias hallii* | 1.88 | [36,38,40,85] |
| *Asclepias hirtella* | 3.16 | [36,38,85] |
| *Asclepias humistrata* | 2.83 | [9,14,37,38,47,70,72,76,78,81,85] |
| *Asclepias incarnata* | 0.63 | [11,36,38,50,62,74,85,91,94,100,102,115] |
| *Asclepias labriformis* | 2.06 | [37,68,115] |
| *Asclepias lanceolata* | 0.33 | [78,85] |
| *Asclepias latifolia* | 2.74 | [38,59,85] |
| *Asclepias lemmonii* | 3.18 | [40,85] |
| *Asclepias linaria* | 5.25 | [37,68,85,115] |
| *Asclepias longifolia* | 2.76 | [78,85] |
| *Asclepias nivea* | 4.59 | [38,40,85,92,115] |
| *Asclepias nyctaginifolia* | 1.91 | [38] |
| *Asclepias obovata* | 0.45 | [85] |
| *Asclepias oenotheroides* | 1.67 | [36,38,85] |
| *Asclepias pedicellata* | 0.26 | [2] |
| *Asclepias perennis* | 5.65 | [36,62,78,85,102,115] |
| *Asclepias pumila* | 0.59 | [38,85] |
| *Asclepias purpurascens* | 0.72 | [36,38,40,85,115] |
| *Asclepias quadrifolia* | 0.61 | [85] |
| *Asclepias rubra* | 0.43 | [85] |
| *Asclepias solanoana* | 1.38 | [40,68,85,115] |
| *Asclepias speciosa* | 1.15* | [36,38,46,50,51,85,87,88,116] |
| *Asclepias stenophylla* | 0.16 | [37] |
| *Asclepias subulata* | 4.04 | [38,85] |
| *Asclepias subverticillata* | 1.14 | [38,85] |
| *Asclepias sullivantii* | 1.76 | [36,38,85] |
| *Asclepias syriaca* | 0.83 | [11,36–40,50–52,59,60,73,85,93,99–101,115,116] |
| *Asclepias texana* | 0.90 | [38,85] |
| *Asclepias tomentosa* | 0.11 | [2,70] |
| *Asclepias tuberosa* | 0.52 | [36,38,85,100] |
| *Asclepias variegata* | 1.19 | [36,85] |
| *Asclepias verticillata* | 0.58 | [36,38,59,70,85,115] |
| *Asclepias vestita* | 7.20 | [40,61,79,85,87] |
| *Asclepias viridiflora* | 0.80 | [38,68,85] |
| *Asclepias viridis* | 3.14 | [36–38,57,66,70,72,85,95,100,115] |
| *Brassica oleracea var. capitata* | 0 | [84] |
| *Calotropis gigantea* | 1.65 | [56] |
| *Calotropis procera* | 2.04 | [87] |
| *Catharanthus roseus* | 0 | [90] |
| *Cynanchum acutum* | 2.81 | [55] |
| *Cynanchum laeve* | 0.87 | [48] |
| *Datura innoxia* | 0 | [97] |
| *Digitalis purpurea* | 0.26 | [63,96] |
| *Funastrum clausum* | 0.38 | [86] |
| *Gomphocarpus cancellatus* | 6.26 | [36,38,85] |
| *Gomphocarpus fruticosus* | 3.94 | [2,36,38,82,85,99] |
| *Gomphocarpus physocarpus* | 3.31 | [2] |
| *Gonolobus suberosus* | 0.10 | [78] |
| *Hibiscus trionum* | 0 | [42] |
| *Ipomoea batatas* | 0 | [65] |
| *Nicandra physalodes* | 0 | [98] |
| *Oenothera biennis* | 0 | [58] |
| *Orthosia scoparia* | 0.14 | [78] |
| *Seutera angustifolia* | 0.27 | [78] |
| *Solanum dulcamara* | 0 | [64] |

*When one non-peer reviewed source [88] (a conference paper) was removed, this value changed from 1.15 mg/g to 1.16 mg/g and did not affect the results of our analyses.

**E Table:** Mean cardenolide polarity and diversity for plant species. Polarity is represented using the index developed by Rasmann and Agrawal 2011 [85] and diversity is expressed as the Shannon-Wiener index applied to HPLC data.

| **Species** | **Polarity** | **Polarity source(s)** | **Diversity** | **Diversity source(s)** |
| --- | --- | --- | --- | --- |
| *Asclepias amplexicaulis* | 15.77 | [85] | 1.76 | [85] |
| *Asclepias angustifolia* | 16.92 | [85] | 1.83 | [85] |
| *Asclepias arenaria* | 15.40 | [85] | 1.60 | [85] |
| *Asclepias asperula* | 16.63 | [85] | 2.34 | [85] |
| *Asclepias barjoniifolia* | 14.39 | [85] | 2.52 | [85] |
| *Asclepias boliviensis* | 17.52 | [85] | 2.02 | [85] |
| *Asclepias brachystephana* | 17.22 | [85] | 2.39 | [85] |
| *Asclepias californica* | 16.04 | [85] | 2.58 | [85] |
| *Asclepias candida* | 15.09 | [85] | 2.47 | [85] |
| *Asclepias cordifolia* | 16.11 | [85] | 2.48 | [85] |
| *Asclepias cryptoceras* | 16.85 | [85] | 1.71 | [85] |
| *Asclepias curassavica* | 9.91 | [51,85] | 1.25 | [50,51,85] |
| *Asclepias engelmanniana* | 11.60 | [85] | 0.81 | [85] |
| *Asclepias eriocarpa* | 16.48 | [85] | 2.23 | [85] |
| *Asclepias erosa* | 17.20 | [85] | 1.46 | [85] |
| *Asclepias exaltata* | 16.48 | [85] | 1.46 | [85] |
| *Asclepias fascicularis* | 16.65 | [85] | 1.43 | [85] |
| *Asclepias hallii* | 16.91 | [85] | 2.08 | [85] |
| *Asclepias hirtella* | 17.36 | [85] | 2.23 | [85] |
| *Asclepias humistrata* | 16.14 | [85] | 2.18 | [85] |
| *Asclepias incarnata* | 12.11 | [51,85] | 1.09 | [50,51,85] |
| *Asclepias lanceolata* | 17.88 | [85] | 1.63 | [85] |
| *Asclepias latifolia* | 17.20 | [85] | 1.68 | [85] |
| *Asclepias lemmonii* | 17.13 | [85] | 1.89 | [85] |
| *Asclepias linaria* | 17.03 | [85] | 2.44 | [85] |
| *Asclepias longifolia* | 17.03 | [85] | 1.98 | [85] |
| *Asclepias nivea* | 17.48 | [85] | 2.47 | [85] |
| *Asclepias obovata* | 17.50 | [85] | 1.03 | [85] |
| *Asclepias oenotheroides* | 17.14 | [85] | 2.15 | [85] |
| *Asclepias perennis* | 16.57 | [85] | 2.63 | [85] |
| *Asclepias pumila* | 13.38 | [85] | 0.49 | [85] |
| *Asclepias purpurascens* | 17.58 | [85] | 1.17 | [85] |
| *Asclepias quadrifolia* | 16.17 | [85] | 1.59 | [85] |
| *Asclepias rubra* | 17.58 | [85] | 1.64 | [85] |
| *Asclepias solanoana* | 16.24 | [85] | 2.18 | [85] |
| *Asclepias speciosa* | 8.82 | [51,85] | 0.66 | [50,51,85] |
| *Asclepias subulata* | 16.54 | [85] | 2.27 | [85] |
| *Asclepias subverticillata* | 15.47 | [85] | 1.59 | [85] |
| *Asclepias sullivantii* | 13.57 | [85] | 1.83 | [85] |
| *Asclepias syriaca* | 10.50 | [51,85] | 1.01 | [51,85] |
| *Asclepias texana* | 17.17 | [85] | 2.15 | [85] |
| *Asclepias tuberosa* | 21.85 | [85] | 0.69 | [85] |
| *Asclepias variegata* | 16.85 | [85] | 1.62 | [85] |
| *Asclepias verticillata* | 20.71 | [85] | 1.06 | [85] |
| *Asclepias vestita* | 17.39 | [85] | 2.16 | [85] |
| *Asclepias viridiflora* | 17.81 | [85] | 1.96 | [85] |
| *Asclepias viridis* | 17.00 | [85] | 2.09 | [85] |
| *Gomphocarpus cancellatus* | 17.42 | [85] | 2.45 | [85] |
| *Gomphocarpus fruticosus* | 17.89 | [85] | 2.05 | [85] |

**F Table:** Mean trichome densities of plant species. Trichome densities are in units of (# sum of adaxial and abaxial surfaces)/mm^2^.

| **Species** | **Trichome density** | **Source(s)** |
| --- | --- | --- |
| *Araujia sericifera* | 891.00 | [106] |
| *Asclepias amplexicaulis* | 0.15 | [35,36,111] |
| *Asclepias angustifolia* | 6.89 | [35,104] |
| *Asclepias arenaria* | 173.20 | [35] |
| *Asclepias asperula* | 3.17 | [36,104] |
| *Asclepias barjoniifolia* | 0.19 | [104] |
| *Asclepias boliviensis* | 27.55 | [35,104] |
| *Asclepias brachystephana* | 154.42 | [35] |
| *Asclepias californica* | 194.04 | [35,36,104] |
| *Asclepias cordifolia* | 0.02 | [35,36,104] |
| *Asclepias cryptoceras* | 2.30 | [35,104] |
| *Asclepias curassavica* | 6.93 | [35,36,62,104] |
| *Asclepias engelmanniana* | 3.66 | [35,104] |
| *Asclepias eriocarpa* | 126.50 | [35,36,104] |
| *Asclepias erosa* | 205.02 | [35,104] |
| *Asclepias exaltata* | 22.85 | [35,36,104] |
| *Asclepias fascicularis* | 0.98 | [36,104] |
| *Asclepias glaucescens* | 3.37 | [35,104] |
| *Asclepias hallii* | 36.77 | [35,36,104] |
| *Asclepias hirtella* | 15.02 | [35,36] |
| *Asclepias humistrata* | 3.50 | [35,104] |
| *Asclepias incarnata* | 8.59 | [36,62,104,105,107] |
| *Asclepias lanceolata* | 7.83 | [35] |
| *Asclepias latifolia* | 31.82 | [104] |
| *Asclepias lemmonii* | 55.02 | [104,111] |
| *Asclepias linaria* | 14.79 | [104,111] |
| *Asclepias longifolia* | 21.58 | [35] |
| *Asclepias nivea* | 28.31 | [35,104] |
| *Asclepias nyctaginifolia* | 13.72 | [35,104] |
| *Asclepias obovate* | 61.64 | [104,111] |
| *Asclepias oenotheroides* | 9.39 | [35,36,104] |
| *Asclepias otarioides* | 353.31 | [35] |
| *Asclepias ovalifolia* | 121.50 | [35] |
| *Asclepias perennis* | 5.29 | [35,36,62,104] |
| *Asclepias pumila* | 3.42 | [104] |
| *Asclepias purpurascens* | 36.50 | [35,36,104] |
| *Asclepias quadrifolia* | 8.00 | [35] |
| *Asclepias rubra* | 6.88 | [35] |
| *Asclepias solanoana* | 297.50 | [35] |
| *Asclepias speciosa* | 92.78 | [35,36,104] |
| *Asclepias subulata* | 29.78 | [35,104,111] |
| *Asclepias subverticillata* | 9.42 | [35,104] |
| *Asclepias sullivantii* | 0.68 | [35,36,104] |
| *Asclepias syriaca* | 95.79 | [35,36,104,107] |
| *Asclepias texana* | 1.27 | [104] |
| *Asclepias tuberosa* | 23.83 | [35,36,104,105,107] |
| *Asclepias variegata* | 4.24 | [36,104,111] |
| *Asclepias verticillata* | 5.07 | [36,104] |
| *Asclepias vestita* | 92.46 | [104,111] |
| *Asclepias viridiflora* | 67.28 | [35,104] |
| *Asclepias viridis* | 19.15 | [35,36,104] |
| *Calotropis procera* | 1177.31 | [112] |
| *Catharanthus roseus* | 0.00 | [114] |
| *Cynanchum laeve* | 21.12 | [107] |
| *Datura innoxia* | 70.60 | [108] |
| *Gomphocarpus cancellatus* | 13.51 | [36,104] |
| *Gomphocarpus fruticosus* | 5.96 | [36,104] |
| *Lycopersicon esculentum* | 80.34 | [109,113] |
| *Vigna unguiculata* | 67.07 | [110,111] |

Full results of generalized linear models testing predictive effects of cardenolide concentration, cardenolide diversity/polarity, and trichome density are presented in G, H, and I Tables (S2 Appendix) respectively.

**G Table:** Results of Generalized linear model testing the predictive effect of mean cardenolide concentration on host performance status. Values are odds ratios with parenthetical standard errors.

| Performance ~ Mean Cardenolide Conc. | |
| --- | --- |
|  | *Dependent variable:* |
|  |  |
|  | Low/High Performance |
|  | |
| Mean Cardenolide Conc. | 1.489^*^ (0.176) |
| Constant | 0.495 (0.414) |
|  | |
| Observations | 64 |
| Log Likelihood | -41.320 |
| Akaike Inf. Crit. | 86.640 |
|  | |
| *Note:* | ^*^p<0.05 ^**^p<0.01 ^***^p<0.001 |

**H Table:** Generalized linear model testing the predictive effects of cardenolide diversity and polarity on host performance status. Values are odds ratios with parenthetical standard errors.

| Performance ~ Diversity + Polarity | |
| --- | --- |
|  | *Dependent variable:* |
|  |  |
|  | Low/High Performance |
|  | |
| Polarity | 1.013 (0.130) |
| Diversity | 0.914 (0.550) |
| Constant | 1.265 (2.048) |
|  | |
| Observations | 49 |
| Log Likelihood | -33.448 |
| Akaike Inf. Crit. | 72.896 |
|  | |
| *Note:* | ^*^p<0.05 ^**^p<0.01 ^***^p<0.001 |

**I Table:** Generalized linear model testing the predictive effects of trichome density on host performance status. Values are odds ratios with parenthetical standard errors.

| Performance ~ Trichome Density | |
| --- | --- |
|  | *Dependent variable:* |
|  |  |
|  | Low/High Performance |
|  | |
| Trichome Density | 1.000 (0.001) |
| Constant | 1.056 (0.283) |
|  | |
| Observations | 59 |
| Log Likelihood | -40.870 |
| Akaike Inf. Crit. | 85.741 |
|  | |
| *Note:* | ^*^p<0.05 ^**^p<0.01 ^***^p<0.001 |

The results of these analyses were identical (at the reported number of digits) to analyses including all sources. Three non-peer reviewed sources provided performance data for *A. meadii*, which we have no cardenolide data for (not included in analyses), one source provided cardenolide values for *Agernatina altissima* (a non-host plant not used in analyses), and one source provided cardenolide values for *A. speciosa*, for which we have 8 other cardenolide sources (its removal changes the value for that species from 1.15 mg/g to 1.16 mg/g; D Table in S2 Appendix).

**Phylogenetic results**

We were unable to obtain reliable results from the phylogenetic ANOVA because of our limited sample size. After trimming our dataset to contain only high and low performance plant species also included in the phylogenetic data provided by Dr. Mark Fishbein, an F test found equal variances for both low and high performance groups (F(21) = 0.717 p = 0.461) and Shapiro-Wilk normality tests showed both groups to be normally distributed (high performance: W = 0.932, p = 0.134, low performance: W = 0.915, p = 0.067) enabling the performance of an ANOVA. However, neither standard nor phylogenetic ANOVAs (100 simulations) found significant differences in cardenolide concentrations between high- and low-performance host plants (F(1, 41) = 1.228, p = 0.274 and F(1, 41) = 1.228, p ≈ 0.3 respectively). Reducing our sample size from 66 to 40 plants for this analysis made group variances statistically similar, groups approximately normally distributed, and reduced the significance of differences in cardenolide concentrations.

We estimated Fritz and Purvis’ D to be 1.027318. This value was not significantly different from the random expectation (D = 1; p = 0.513) but was significantly different from the Brownian expectation (D = 0; p = 0.023). Similarly, we calculated Blomberg’s K to be 0.271091, which was not significantly different from K calculated on the same dataset after 1000 randomizations (p ≈ 0.08). Therefore, we fail to show that either trait has a significant phylogenetic signal (B Fig in S2 Appendix).

**B Fig:** Phylogenetic tree showing host status and cardenolide concentrations of plant species included in the phylogenetic ANOVA (n = 40).

**References**

1. Robertson GF, Zalucki MP, Paine TD. Larval Host Choice of the Monarch Butterfly (Danaus plexippus L.) on Four Native California Desert Milkweed Species. J Insect Behav. 2015;28: 582–592. doi:10.1007/s10905-015-9524-2

2. Malcolm SB. Cardenolide-Mediated Interactions between Plants and Herbivores. 2nd ed. In: Rosenthal G, Berenbaum M, editors. Herbivores: Their Interactions with Secondary Plant Metabolites. 2nd ed. Elsevier Inc.; 1991. pp. 251–296.

3. Jones PL, Petschenka G, Flacht L, Agrawal AA. Cardenolide Intake, Sequestration, and Excretion by the Monarch Butterfly along Gradients of Plant Toxicity and Larval Ontogeny. J Chem Ecol. 2019;45: 264–277. doi:10.1007/s10886-019-01055-7

4. Zalucki MP, Clarke AR, Malcolm SB. Ecology and Behavior of First Instar Larval Lepidoptera. Annual Review of Entomology. 2002;47: 361–393. doi:10.1146/annurev.ento.47.091201.145220

5. Agrawal AA, Fishbein M, Jetter R, Salminen J-P, Goldstein JB, Freitag AE, et al. Phylogenetlc Ecology of Leaf Surface Traits in the Milkweeds (Asclepias spp.): Chemistry, Ecophysiology, and Insect Behavior. The New Phytologist. 2009;183: 848–867.

6. Malcolm SB. Milkweeds, monarch butterflies and the ecological significance of cardenolides. Chemoecology. 1994;5: 101–117. doi:10.1007/BF01240595

7. Hulley PE. Caterpillar attacks plant mechanical defence by mowing trichomes before feeding. Ecological Entomology. 1988;13: 239–241. doi:https://doi.org/10.1111/j.1365-2311.1988.tb00351.x

8. Faldyn MJ, Hunter MD, Elderd BD. Climate change and an invasive, tropical milkweed: an ecological trap for monarch butterflies. Ecology. 2018;99: 1031–1038. doi:https://doi.org/10.1002/ecy.2198

9. Zalucki MP, Brower LP, Alonso‐M A. Detrimental effects of latex and cardiac glycosides on survival and growth of first-instar monarch butterfly larvae Danaus plexippus feeding on the sandhill milkweed Asclepias humistrata. Ecological Entomology. 2001;26: 212–224. doi:10.1046/j.1365-2311.2001.00313.x

10. Zalucki MP, Brower LP. Survival of first instar larvae of Danaus plexippus (Lepidoptera: Danainae) in relation to cardiac glycoside and latex content of Asclepias humistrata (Asclepiadaceae). Chemoecology. 1992;3: 81–93. doi:10.1007/BF01245886

11. Tao L, Berns AR, Hunter MD. Why does a good thing become too much? Interactions between foliar nutrients and toxins determine performance of an insect herbivore. Functional Ecology. 2014;28: 190–196. doi:https://doi.org/10.1111/1365-2435.12163

12. Zalucki MP, Brower LP, Malcolm SB. Oviposition by Danaus plexippus in relation to cardenolide content of three Asclepias species in the southeastern U.S.A. Ecol Entomol. 1990;15: 231–240. doi:10.1111/j.1365-2311.1990.tb00804.x

13. Rasmann S, Johnson MD, Agrawal AA. Induced Responses to Herbivory and Jasmonate in Three Milkweed Species. J Chem Ecol. 2009;35: 1326–1334. doi:10.1007/s10886-009-9719-0

14. Zalucki MP, Brower LP, Malcolm SB. Oviposition by Danaus plexippus in relation to cardenolide content of three Asclepias species in the southeastern U.S.A. Ecol Entomol. 1990;15: 231–240. doi:10.1111/j.1365-2311.1990.tb00804.x

15. Glassmire AE, Zehr LN, Wetzel WC. Disentangling dimensions of phytochemical diversity: alpha and beta have contrasting effects on an insect herbivore. Ecology. 2020;101: e03158. doi:10.1002/ecy.3158

16. Detzel A, Wink M. Evidence for a Cardenolide Carrier in Oncopeltus fasciatus (Dallas) (Insecta: Hemiptera). Zeitschrift für Naturforschung C. 1995;50: 127–134. doi:10.1515/znc-1995-1-219

17. Richards LA, Glassmire AE, Ochsenrider KM, Smilanich AM, Dodson CD, Jeffrey CS, et al. Phytochemical diversity and synergistic effects on herbivores. Phytochem Rev. 2016;15: 1153–1166. doi:10.1007/s11101-016-9479-8

18. Jones CG, Firn RD, Malcolm SB, Chaloner WG, Harper JL, Lawton JH. On the evolution of plant secondary chemical diversity. Philosophical Transactions of the Royal Society of London Series B: Biological Sciences. 1991;333: 273–280. doi:10.1098/rstb.1991.0077

19. Khabsa M, Giles CL. The Number of Scholarly Documents on the Public Web. PLOS ONE. 2014;9: e93949. doi:10.1371/journal.pone.0093949

20. Harzing AW. Publish or Perish. 2007. Available: https://harzing.com/resources/publish-or-perish

21. RStudio Team. RStudio: Integrated Development Environment for R. Boston, MA: RStudio, PBC; 2021. Available: http://www.rstudio.com/

22. R Core Team. R: A Language and Environment for Statistical Computing. Vienna, Austria: R Foundation for Statistical Computing; 2021. Available: https://www.R-project.org/

23. Wickham H, Averick M, Bryan J, Chang W, McGowan LD, François R, et al. Welcome to the Tidyverse. Journal of Open Source Software. 2019;4: 1686. doi:10.21105/joss.01686

24. Kassambara A. ggpubr: “ggplot2” Based Publication Ready Plots. 2020. Available: https://CRAN.R-project.org/package=ggpubr

25. Revell LJ. phytools: An R package for phylogenetic comparative biology (and other things). Methods in Ecology and Evolution. 2012;3: 217–223.

26. Paradis E, Schliep K. ape 5.0: an environment for modern phylogenetics and evolutionary analyses in R. Bioinformatics. 2019;35: 526–528.

27. Wilke CO. cowplot: Streamlined Plot Theme and Plot Annotations for “ggplot2.” 2020. Available: https://CRAN.R-project.org/package=cowplot

28. Bates D, Maechler M, Bolker B, Walker S. Fitting Linear Mixed-Effects Models Using lme4. Journal of Statistical Software. 2015;67: 1–48. doi:10.18637/jss.v067.i01.

29. Lenth RV. emmeans: Estimated Marginal Means, aka Least-Squares Means. 2021. Available: https://CRAN.R-project.org/package=emmeans

30. Therneau TM, Grambsch PM. Modeling Survival Data: Extending the Cox Model. New York: Springer; 2000.

31. Kassambara A, Kosinski M, Biecek P. survminer: Drawing Survival Curves using “ggplot2.” 2020. Available: https://CRAN.R-project.org/package=survminer

32. Therneau TM. coxme: Mixed Effects Cox Models. 2020. Available: https://CRAN.R-project.org/package=coxme

33. Orme D, Freckleton R, Thomas G, Petzoldt T, Fritz S, Isaac N, et al. caper: Comparative Analyses of Phylogenetics and Evolution in R. 2018. Available: https://CRAN.R-project.org/package=caper

34. Hlavac M. stargazer: Well-Formatted Regression and Summary Statistics Tables. Bratislava, Slovakia: Central European Labour Studies Institute (CELSI); 2018. Available: https://CRAN.R-project.org/package=stargazer

35. Fishbein M, Straub SCK, Boutte J, Hansen K, Cronn RC, Liston A. Evolution at the tips: Asclepias phylogenomics and new perspectives on leaf surfaces. American Journal of Botany. 2018;105: 514–524. doi:https://doi.org/10.1002/ajb2.1062

36. Agrawal AA, Fishbein M. Plant Defense Syndromes. Wiley; 2016. doi:10.6084/m9.figshare.c.3299276.v1

37. Agrawal AA, Hastings AP. Trade-offs constrain the evolution of an inducible defense within but not between plant species. Ecology. 2019;100: e02857. doi:https://doi.org/10.1002/ecy.2857

38. Agrawal AA, Salminen J-P, Fishbein M. Phylogenetic Trends in Phenolic Metabolism of Milkweeds (Asclepias): Evidence for Escalation. Evolution. 2009;63: 663–673. doi:https://doi.org/10.1111/j.1558-5646.2008.00573.x

39. Agrawal AA, Patrick ET, Hastings AP. Tests of the coupled expression of latex and cardenolide plant defense in common milkweed (Asclepias syriaca). Ecosphere. 2014;5: art126. doi:https://doi.org/10.1890/ES14-00161.1

40. Agrawal AA, Rasmann S, Fishbein M. Macroevolutionary Trends in the Defense of Milkweeds against Monarchs. 1st ed. Monarchs in a Changing World: Biology and Conservation of an Iconic Butterfly. 1st ed. Cornell University Press; 2015. Available: https://www.jstor.org/stable/10.7591/j.ctt20fw696

41. Agrawal AA, Böröczky K, Haribal M, Hastings AP, White RA, Jiang R-W, et al. Cardenolides, toxicity, and the costs of sequestration in the coevolutionary interaction between monarchs and milkweeds. PNAS. 2021;118. doi:10.1073/pnas.2024463118

42. Ateya A-M, Sayed ZIE, Fekr M. Chemical Constituents, Cytotoxicity, Anti-oxidant, Hypoglycemic and Anti- hypertensive Activities of Egyptian Hibiscus trionum. Australian Journal of Basic and Applied Sciences. 2012;6: 756–766.

43. Bolsinger M, Lier ME, Lansky DM, Hughes PR. Influence of Ozone Air Pollution on Plant-Herbivore Interactions. Part 1: Biochemical Changes in Ornamental Milkweed (Asclepias curassa rica L.; Asclepiadaceae) Induced by Ozone. Environmental Pollution. 1991;72: 15.

44. Brower LP, Seiber JN, Nelson CJ, Lynch SP, Tuskes PM. Plant-determined variation in the cardenolide content, thin-layer chromatography profiles, and emetic potency of monarch butterflies, Danaus plexippus reared on the milkweed, Asclepias eriocarpa in California. J Chem Ecol. 1982;8: 579–633. doi:10.1007/BF00989631

45. Brower LP, Seiber JN, Nelson CJ, Lynch SP, Hoggard MP, Cohen JA. Plant-determined variation in cardenolide content and thin-layer chromatography profiles of monarch butterflies, Danaus plexippus reared on milkweed plants in California: 3. Asclepias californica. J Chem Ecol. 1984;10: 1823–1857. doi:10.1007/BF00987364

46. Brower LP, Seiber JN, Nelson CJ, Lynch SP, Holland MM. Plant-determined variation in the cardenolide content, thin-layer chromatography profiles, and emetic potency of monarch butterflies, Danaus plexippus L. Reared on milkweed plants in California: 2. Asclepias speciosa. J Chem Ecol. 1984;10: 601–639. doi:10.1007/BF00994224

47. Cohen JA, Brower LP. Oviposition and Larval Success of Wild Monarch Butterflies (Lepidoptera: Danaidae) in Relation to Host Plant Size and Cardenolide Concentration. Journal of the Kansas Entomological Society. 1982;55: 343–348.

48. Colvin SM, Snyder JC, Thacker R, Yeargan KV. Thinking outside the Asclepias Box: Oleander Aphids and Honeyvine Milkweed. Ann Entomol Soc Am. 2013;106: 214–221. doi:10.1603/AN11189

49. Dall’Acqua S, Bolego C, Cignarella A, Gaion RM, Innocenti G. Vasoprotective activity of standardized Achillea millefolium extract. Phytomedicine. 2011;18: 1031–1036. doi:10.1016/j.phymed.2011.05.005

50. Decker LE, De Roode JC, Hunter MD. Data from: Elevated atmospheric concentrations of carbon dioxide reduce monarch tolerance and increase parasite virulence by altering the medicinal properties of milkweeds. Dryad; 2019. p. 23137 bytes. doi:10.5061/DRYAD.D68KG81

51. Decker LE, Soule AJ, De Roode JC, Hunter MD. Data from: Phytochemical changes in milkweed induced by elevated CO2 alter wing morphology but not toxin sequestration in monarch butterflies. Dryad; 2019. p. 102422 bytes. doi:10.5061/DRYAD.MK3TJ78

52. DeLaMater DS, Couture JJ, Puzey JR, Dalgleish HJ. Range-wide variations in common milkweed traits and their effect on monarch larvae. American Journal of Botany. 2021;108: 388–401. doi:https://doi.org/10.1002/ajb2.1630

53. Couture JJ, Serbin SP, Townsend PA. Spectroscopic sensitivity of real-time, rapidly induced phytochemical change in response to damage. New Phytologist. 2013;198: 311–319. doi:10.1111/nph.12159

54. Dobler S, Daloze D, Pasteels JM. Sequestration of plant compounds in a leaf beetle’s defensive secretion: cardenolides in Chrysochus. Chemoecology. 1998;8: 111–118. doi:10.1007/s000490050015

55. El-Bakry AA, Genady E, Ghazi SM, Rafat SA. Regeneration, Cardenolide and Flavonoid Production from In Vitro Cultures of Cynanchum acutum L. (Asclepiadaceae). Australian Journal of Basic and Applied Sciences. 2011;5: 15.

56. Erdman MD. Nutrient and cardenolide composition of unextracted and solvent-extracted Calotropis procera. J Agric Food Chem. 1983;31: 509–513. doi:10.1021/jf00117a012

57. George G. Extraction and Quantification of Cardiac Glycosides from Asclepias viridis Leaves. M.S., Texas A&M University - Commerce. 2020. Available: https://search.proquest.com/docview/2411089349/abstract/FC57C7935B594C8CPQ/1

58. Granica S, Czerwińska ME, Piwowarski JP, Ziaja M, Kiss AK. Chemical Composition, Antioxidative and Anti-Inflammatory Activity of Extracts Prepared from Aerial Parts of Oenothera biennis L. and Oenothera paradoxa Hudziok Obtained after Seeds Cultivation. J Agric Food Chem. 2013;61: 801–810. doi:10.1021/jf304002h

59. Hoang K, Tao L, Hunter MD, de Roode JC. Host Diet Affects the Morphology of Monarch Butterfly Parasites. Journal of Parasitology. 2017;103: 228–236. doi:10.1645/16-142

60. Isman MB, Duffey SS, Scudder GGE. Cardenolide content of some leaf- and stem-feeding insects on temperate North American milkweeds (Asclepias spp.). Canadian Journal of Zoology. 1977 [cited 21 Apr 2021]. doi:10.1139/z77-130

61. Isman MB, Duffey SS, Scudder GGE. Variation in cardenolide content of the lygaeid bugs, Oncopeltus fasciatus and Lygaeus kalmii kalmii and of their milkweed hosts (Asclepias spp.) in central California. J Chem Ecol. 1977;3: 613–624. doi:10.1007/BF00988061

62. Jones PL, Agrawal AA. Data from: Beyond preference and performance: host plant selection by monarch butterflies, Danaus plexippus. Dryad; 2019. p. 77143 bytes. doi:10.5061/DRYAD.8HD6764

63. Kairuz E, Pérez-Alonso N, Capote-Pérez A, Pérez-Pérez A, Espinosa-Antón AA, Angenon G, et al. Enhancement of cardenolide production in transgenic Digitalis purpurea L. by expressing a progesterone-5β-reductase from Arabidopsis thaliana L. Industrial Crops and Products. 2020;146: 112166. doi:10.1016/j.indcrop.2020.112166

64. Lee Y-Y, Hashimoto F, Yahara S, Nohara T, Yoshida N. Steroidal Glycosides from Solanum dulcamara. Chemical & Pharmaceutical Bulletin. 1994;42: 707–709. doi:10.1248/cpb.42.707

65. Lee C-L, Lee S-L, Chen C-J, Chen H-C, Kao M-C, Liu C-H, et al. Characterization of Secondary Metabolites from Purple Ipomoea batatas Leaves and Their Effects on Glucose Uptake. Molecules. 2016;21: 745. doi:10.3390/molecules21060745

66. Lynch SP, Martin RA. Cardenolide Content and Thin-Layer Chromatography Profiles of Monarch Butterflies, Danaus plexippus L., and their Larval Host-Plant Milkweed, Asclepias viridis Walt., in Northwestern Louisiana. Journal of Chemical Ecology. 1987;13: 24.

67. Malcolm SB. Chemical defence in chewing and sucking insect herbivores: Plant-derived cardenolides in the monarch butterfly and oleander aphid. Chemoecology. 1990;1: 12–21. doi:10.1007/BF01240581

68. Roeske CN, Seiber JN, Brower LP, Moffitt CM. Milkweed Cardenolides and Their Comparative Processing by Monarch Butterflies (Danaus plexippus L.). In: Wallace JW, Mansell RL, editors. Biochemical Interaction Between Plants and Insects. Boston, MA: Springer US; 1976. pp. 93–167. doi:10.1007/978-1-4684-2646-5_3

69. Seiber JN, Lee SM, Benson JM. Cardiac Glycosides (Cardenolides) in Species of Asclepias (Asclepidaceae). In: Keeler RF, Tu AT, editors. Handbook of Natural Toxins. New York: CRC Press; 1983. pp. 43–83.

70. Malcolm SB, Brower LP. Selective Oviposition by Monarch Butterflies (Danaus plexippus L.) in a Mixed Stand of Asclepias curassavica L. and A. incarnata L. in South Florida. Journal of the Lepidopterists’ Society. 1986;40: 255–263.

71. Malcolm SB, Brower LP. Evolutionary and Ecological Implications of Cardenolide Sequestration in the Monarch Butterfly. Experientia. 1989;45: 284–295. doi:10.1007/BF01951814

72. Malcolm SB, Cockrell BJ, Brower LP. Spring recolonization of eastern North America by the monarch butterfly: successive brood or single sweep migration? In: Malcolm SB, Zalucki MP, editors. Biology and conservation of the monarch butterfly. Los Angeles, Calif: Natural History Museum of Los Angeles Co; 1988. pp. 253–267.

73. Malcolm SB, Cockrell BJ, Brower LP. Cardenolide fingerprint of monarch butterflies reared on common milkweed,Asclepias syriaca L. J Chem Ecol. 1989;15: 819–853. doi:10.1007/BF01015180

74. Martel JW, Malcolm SB. Density-Dependent Reduction and Induction of Milkweed Cardenolides by a Sucking Insect Herbivore. J Chem Ecol. 2004;30: 545–561. doi:10.1023/B:JOEC.0000018628.48604.79

75. Martin RA, Lynch SP. Cardenolide Content and Thin-Layer Chromatography Profiles of Monarch Butterflies, Danaus plexippus L., and Their Larval Host-Plant Milkweed, Asclepias asperula subsp. capricornu (Woods.) Woods., in North Central Texas. Journal of Chemical Ecology. 1988;14: 24.

76. Martin RA, Lynch SP, Brower LP, Malcolm SB, Van Hook T. Cardenolide content, emetic potency, and thin-layer chromatography profiles of monarch butterflies, Danaus plexippus, and their larval host-plant milkweed, Asclepias humistrata, in Florida. Chemoecology. 1992;3: 1–13. doi:10.1007/BF01261450

77. May P. A Phytochemical Investigation of the Toxic Plant Eupatorium rugosum. Thesis, Georgia Institute of Technology. 1978. Available: https://smartech.gatech.edu/bitstream/handle/1853/27302/may_peter_e_197812_ms_135713.pdf

78. Moranz R. Geographic and temporal variation of the cardenolide-based chemical de- fenses of the queen butterfly in relation to host plant cardenolides. M.S., University of Florida. 1996.

79. Nelson CJ. A model for cardenolide and cardenolide glycoside storage by the monarch butterfly, Danaus plexippus (L.). In: Malcolm SB, Zalucki MP, editors. Biology and conservation of the monarch butterfly. Los Angeles, Calif: Natural History Museum of Los Angeles Co; 1988. pp. 83–90.

80. Nelson CJ, Seiber JN, Brower LP. Seasonal and intraplant variation of cardenolide content in the California milkweed, Asclepias eriocarpa, and implications for plant defense. J Chem Ecol. 1981;7: 981–1010. doi:10.1007/BF00987622

81. Nishio S, Blum M, Takahashi S. Intraplant Distribution of Cardenolides in Asclepias Humistrata (Asclepiadaceae), With Additional Notes on Their Fates In Tetraopes melanurus (Coleoptera: Cerambycidae) and Rhyssomatus lineaticollis (Coleoptera: Curculionidae). Memoirs of the College of Agriculture, Kyoto University. 1983;122: 43–52.

82. Oyeyele SO, Zalucki MP. Cardiac glycosides and oviposition by Danaus plexippus on Asclepias fruticosa in south-east Queensland (Australia), with notes on the effect of plant nitrogen content. Ecol Entomol. 1990;15: 177–185. doi:10.1111/j.1365-2311.1990.tb00799.x

83. Palomino-Schätzlein M, Montaño MC, Escrig PV, Boira H, Corma A, Pineda-Lucena A, et al. Identification of Bioactive Compounds in Polar and Nonpolar Extracts of Araujia sericifera. Planta Medica International Open. 2017;4: e93–e103. doi:10.1055/s-0043-121151

84. Park S, Valan Arasu M, Lee M-K, Chun J-H, Seo JM, Lee S-W, et al. Quantification of glucosinolates, anthocyanins, free amino acids, and vitamin C in inbred lines of cabbage (Brassica oleracea L.). Food Chemistry. 2014;145: 77–85. doi:10.1016/j.foodchem.2013.08.010

85. Rasmann S, Agrawal AA. Latitudinal patterns in plant defense: evolution of cardenolides, their toxicity and induction following herbivory. Ecology Letters. 2011;14: 476–483. doi:10.1111/j.1461-0248.2011.01609.x

86. Ritland DB. Palatability of Aposematic Queen Butterflies (Danaus gilippus) Feeding on Sarcostemma clausum (Asclepiadaceae) in Florida. J Chem Ecol. 1991;17: 1593–1610. doi:10.1007/BF00984691

87. Seiber JN, Nelson CJ, Lee SM. Cardenolides in the latex and leaves of seven Asclepias species and Calotropis procera. Phytochemistry. 1982;21: 2343–2348. doi:10.1016/0031-9422(82)85202-3

88. Seiber JN, Lee SM, McChesney MM, Watson TR, Nelson CJ, Brower LP. New Cardiac Glycosides (Cardenolides) From Asclepias Species. Plant Toxicity: Proceedings of the Australia-USA Poisonous Plants Symposium, Brisbane, Australia. Queensland, Australia: The Queensland Poisonous Plants Committee; 1985. pp. 427–437.

89. Seiber JN, Brower LP, Lee SM, McChesney MM, Cheung HTA, Nelson CJ, et al. Cardenolide connection between overwintering monarch butterflies from Mexico and their larval food plant,Asclepias syriaca. J Chem Ecol. 1986;12: 1157–1170. doi:10.1007/BF01639002

90. Siddiqui MJ, Ismail Z, Saidan N. Simultaneous determination of secondary metabolites from Vinca rosea plant extractives by reverse phase high performance liquid chromatography. Phcog Mag. 2011;7: 92. doi:10.4103/0973-1296.80662

91. Sternberg ED, De Roode JC, Hunter MD. Trans-generational parasite protection associated with paternal diet. Journal of Animal Ecology. 2015;84: 310–321. doi:10.1111/1365-2656.12289

92. Tahsler BD. The distribution of cardenolides in Asclepias curassavica and A. nivea and its effect on the uptake of cardenolide dynamics of natural monarch populations. B.A., Amherst College. 1975.

93. Tan W-H, Tao L, Hoang KM, Hunter MD, De Roode JC. Data from: The effects of milkweed induced defense on parasite resistance in monarch butterflies, Danaus plexippus. Dryad; 2019. p. 24338 bytes. doi:10.5061/DRYAD.3PF346V

94. Tan W-H, Acevedo T, Harris E, Alcaide T, Walters J, Hunter M, et al. Data from: Transcriptomics of monarch butterflies (Danaus plexippus) reveals that toxic host plants alter expression of detoxification genes and down-regulate a small number of immune genes. Dryad; 2019. p. 32837 bytes. doi:10.5061/DRYAD.53T43DM

95. Van Hook T, Zalucki MP. Oviposition by Danaus Plexippus (Nymphalidae: Danainae) on Asclepias viridis in Northern Florida. Journal of the Lepidopterists’ Society. 1991;45: 7.

96. Verma SK, Gantait S, Jeong BR, Hwang SJ. Enhanced growth and cardenolides production in Digitalis purpurea under the influence of different LED exposures in the plant factory. Sci Rep. 2018;8: 18009. doi:10.1038/s41598-018-36113-9

97. Witte L, Müller K, Arfmann HA. Investigation of the Alkaloid Pattern of Datura innoxia Plants by Capillary Gas-Liquid-Chromatography-Mass Spectrometry. Planta Med. 1987;53: 192–197. doi:10.1055/s-2006-962670

98. Yu M-Y, Zhao G-T, Liu J-Q, Khan A, Peng X-R, Zhou L, et al. Withanolides from aerial parts of Nicandra physalodes. Phytochemistry. 2017;137: 148–155. doi:10.1016/j.phytochem.2017.02.009

99. Zalucki MP, Oyeyele S, Vowles P. Selective Oviposition by Danaus Plexippus (l.) (lepidoptera: Nymphalidae) in a Mixed Stand of Asclepias Fruticosa and a. Curassavica in Southeast Queensland. Australian Journal of Entomology. 1989;28: 141–146. doi:https://doi.org/10.1111/j.1440-6055.1989.tb01211.x

100. Zehnder CB, Hunter MD. Interspecific Variation Within the Genus Asclepias in Response to Herbivory by a Phloem-feeding Insect Herbivore. J Chem Ecol. 2007;33: 2044–2053. doi:10.1007/s10886-007-9364-4

101. Züst T, Agrawal AA. Data from: Population growth and sequestration of plant toxins along a gradient of specialization in four aphid species on the common milkweed Asclepias syriaca. Dryad; 2016. p. 344093 bytes. doi:10.5061/DRYAD.35NN0

102. Züst T, Mou S, Agrawal AA. Data from: What doesn’t kill you makes you stronger: the burdens and benefits of toxin sequestration in a milkweed aphid. Dryad; 2018. p. 66329 bytes. doi:10.5061/DRYAD.3512GH3

103. Züst T, Petschenka G, Hastings AP, Agrawal AA. Toxicity of Milkweed Leaves and Latex: Chromatographic Quantification Versus Biological Activity of Cardenolides in 16 Asclepias Species. J Chem Ecol. 2019;45: 50–60. doi:10.1007/s10886-018-1040-3

104. Agrawal AA, Fishbein M. Phylogenetic escalation and decline of plant defense strategies. Proceedings of the National Academy of Sciences. 2008;105: 10057–10060. doi:10.1073/pnas.0802368105

105. Baker AM, Redmond CT, Malcolm SB, Potter DA. Suitability of native milkweed (Asclepias) species versus cultivars for supporting monarch butterflies and bees in urban gardens. PeerJ. 2020;8: e9823. doi:10.7717/peerj.9823

106. Carvalho R, Pellissari LCO, Pace MR, Scremin-Dias E, De Oliveira Arruda R, Farinaccio MA. Leaf morphoanatomy of Araujia and Morrenia (Asclepiadoideae, Apocynaceae): phylogenetic implications and species key. Botanical Journal of the Linnean Society. 2017;183: 280–293. doi:10.1093/botlinnean/bow004

107. Colvin SM. Tritrophic effects of milkweed species on natural enemies of Aphis nerii. Ph.D., University of Kentucky. 2011. Available: https://www.proquest.com/docview/1507564407/abstract/D92B039CBEC34CB6PQ/1

108. Gómez-Nucamendi O, Hernandez-Sandoval L, Figueroa M, Martínez M. Leaf anatomy of four species and one variety of Datura (Solanoidea, Solanaceae). Journal of Plant Biology Research. 2016;5: 34–47.

109. Maluf WR, Inoue IF, Ferreira R de PD, Gomes LAA, Castro EM de, Cardoso M das G. Higher glandular trichome density in tomato leaflets and repellence to spider mites. Pesq agropec bras. 2007;42: 1227–1235. doi:10.1590/S0100-204X2007000900003

110. Oghiakhe S. Effect of pubescence in cowpea resistance to the legume pod borer Maruca testulalis (Lepidoptera: Pyralidae). Crop Protection. 1995;14: 379–387. doi:10.1016/0261-2194(95)00018-H

111. Oghiakhe S, Jackai LEN, Makanjuola WA, Hodgson CJ. Morphology, distribution, and the role of trichomes in cowpea (Vigna unguiculata) resistance to the legume pod borer, Maruca testulalis (Lepidoptera: Pyralidae). Bulletin of Entomological Research. 1992;82: 499–505. doi:10.1017/S0007485300042577

112. Rivas R, Barros V, Falcão H, Frosi G, Arruda E, Santos M. Ecophysiological Traits of Invasive C3 Species Calotropis procera to Maintain High Photosynthetic Performance Under High VPD and Low Soil Water Balance in Semi-Arid and Seacoast Zones. Front Plant Sci. 2020;11. doi:10.3389/fpls.2020.00717

113. Simmons AT, Gurr GM, McGrath D, Nicol HI, Martin PM. Trichomes of Lycopersicon spp. and their effect on Myzus persicae (Sulzer) (Hemiptera: Aphididae). Australian Journal of Entomology. 2003;42: 373–378. doi:https://doi.org/10.1046/j.1440-6055.2003.00376.x

114. Verma V, Chandra N. Biochemical and Ultrastructural Changes in Sida cordifolia L. and Catharanthus roseus L. to Auto Pollution. International Scholarly Research Notices. 2014;2014: 1–11. doi:10.1155/2014/263092

115. Züst T, Petschenka G, Hastings AP, Agrawal AA. Toxicity of Milkweed Leaves and Latex: Chromatographic Quantification Versus Biological Activity of Cardenolides in 16 Asclepias Species. J Chem Ecol. 2019;45: 50–60. doi:10.1007/s10886-018-1040-3

116. Seiber JN, Brower LP, Lee SM, McChesney MM, Cheung HTA, Nelson CJ, et al. Cardenolide connection between overwintering monarch butterflies from Mexico and their larval food plant, Asclepias syriaca. J Chem Ecol. 1986;12: 1157–1170. doi:10.1007/BF01639002
