# Supplementary material for: Host plant specificity of the monarch butterfly Danaus plexippus: A systematic review and meta-analysis
Source: PLoS One. 2022 Jun 14;17(6):e0269701. doi: 10.1371/journal.pone.0269701 (PMC9197062; doi:10.1371/journal.pone.0269701)
Supplement: S3 Appendix — Includes supplemental methods and model details. (A-F Tables). (DOCX) [file pone.0269701.s003.docx]

**S3 Appendix: Experimental supplement**

**Supplemental methods**

Larvae were not reared to adulthood in all experimental trials due to limited availability of plant tissues. Details of all four experimental trials are presented in A Table in S3 Appendix. Different family lines were used in trials because of the difficulty in getting the same pair to mate repeatedly in a relatively short period of time. To reduce genetic variation, many of the same individuals were used in different trials but with different mates. Although it is possible that this variation has affected our results, we minimize this risk by including trial number (a proxy of family line) as a random effect in our statistical analyses (C-E Tables in S3 Appendix).

**A Table:** Details of No-choice feeding assays

| **Trial** | **Start Date** | **Family Line** | **Species Tested** | **Larval Fate** |
| --- | --- | --- | --- | --- |
| 1 | 07-22-20 | LC | *N. oleander*, *B. oleracea var capitata*, & *A. curassavica* | Survivors reared to adulthood |
| 2 | 10-15-20 | CC | *S. dulcamara, A. sericifera,* & *A. curassavica* | Survivors reared to adulthood |
| 3 | 10-20-20 | CC | *G. suberosus, A. sericifera,* & *A. curassavica* | Survivors reared to adulthood |
| 4 | 01-15-21 | WC6xCC | *A. sericifera* & *A. curassavica* | Survivors reared until plants exhausted (~day 18) |

All statistical analyses were conducted using RStudio 2021.09.0+351 "Ghost Orchid" Release for macOS [1] running R version 4.1.1 [2]. R scripts are available upon request. The following R packages were used: tidyverse [3], cowplot [4], lme4 [5], emmeans [6], survival [7], survminer [8], coxme [9], and stargazer [10].

**Supplemental results**

Full results of the Cox proportional hazard model, generalized linear model of survival on days 1-5, and generalized linear model of survival on days 6-30 are presented in C-E Tables in S3 Appendix.

**B Table:** Summary of the Cox proportional hazard model of larval survival on different species of plant. LR Test = likelihood ratio $\chi^{2}$test

|  | |
| --- | --- |
|  | *Dependent variable:* |
|  |  |
|  | Day |
|  | |
| A. curassavica | -3.732^***^ (0.454) |
| A. sericifera | -0.928^***^ (0.241) |
| G. suberosus | -1.607^***^ (0.431) |
|  |  |
|  | |
| Observations | 208 |
| R^2^ | 0.457 |
| Max. Possible R^2^ | 0.999 |
| Log Likelihood | -663.635 |
| Wald Test | 69.680^***^ (df = 3) |
| LR Test | 127.442^***^ (df = 3) |
| Score (Logrank) Test | 109.391^***^ (df = 3) |
|  | |
| *Note:* | ^*^p<0.1 ^**^p<0.05 ^***^p<0.01 |

**C Table:** Generalized linear mixed model testing the predictive effect of species on survival to day five. Values are estimates with parenthetical standard errors.

| Survived to Day 5 ~ Species + (1\|Trial) + (1\|Larval ID) | |
| --- | --- |
|  | Dependent variable: |
|  |  |
|  | Survived to Day 5 |
|  | |
| A. sericifera | -4.563^***^ (1.053) |
| G. suberosus | -2.251 (1.246) |
| Constant | 2.944^**^ (1.026) |
|  | |
| Observations | 156 |
| Log Likelihood | -66.651 |
| Akaike Inf. Crit. | 143.302 |
| Bayesian Inf. Crit. | 158.552 |
|  | |
| Note: | ^*^p<0.05 ^**^p<0.01 ^***^p<0.001 |

**D Table:** Generalized linear mixed model testing the predictive effect of species on survival to day thirty. Larvae dead at day five were excluded from this model. Values are estimates with parenthetical standard errors.

| Survived to Day 30 ~ Species + (1\|Trial) + (1\|Larval ID) | |
| --- | --- |
|  | Dependent variable: |
|  |  |
|  | Survived to Day 30 |
|  | |
| A. sericifera | -4.391^***^ (1.131) |
| G. suberosus | -3.750 ^**^ (1.378) |
| Constant | 2.140^**^ (0.774) |
|  | |
| Observations | 46 |
| Log Likelihood | -15.701 |
| Akaike Inf. Crit. | 41.402 |
| Bayesian Inf. Crit. | 50.525 |
|  | |
| Note: | ^*^p<0.05 ^**^p<0.01 ^***^p<0.001 |

Full results for the linear mixed model of larval development are presented in E Table in S3 Appendix, and the results of pairwise comparisons of model estimated marginal means are presented in F Table in S3 Appendix.

**E Table:** Linear mixed model of larval development curves. Values are logits with parenthetical standard errors.

| Mean Instar ~ Day + Species + (1\|Trial) + (1 + Day\|Larval ID) | |
| --- | --- |
|  | Dependent variable: |
|  |  |
|  | Instar |
|  | |
| Day | 0.217^***^ (0.006) |
| A. curassavica | 0.552^***^ (0.052) |
| G. suberosus | 0.291^*^ (0.121) |
| Constant | 1.326^***^ (0.371) |
|  | |
| Observations | 1,455 |
| Log Likelihood | -1,342.143 |
| Akaike Inf. Crit. | 2,702.287 |
| Bayesian Inf. Crit. | 2,749.831 |
|  | |
| Note: | ^*^p<0.05 ^**^p<0.01 ^***^p<0.001 |

**F Table:** Pairwise comparisons of the estimated marginal means (Tukey) for each plant species in the linear mixed model of larval development curves (E Table in S3 Appendix).

| **Species Comparison** | **Estimate** | **SE** | **df** | **t ratio** | **p value** |
| --- | --- | --- | --- | --- | --- |
| *A. sericifera - A. curassavica* | -0.552 | 0.0524 | 134.8 | -10.551 | <0.0001 |
| *A. sericifera - G. suberosus* | -0.291 | 0.1241 | 101.4 | -2.343 | 0.0544 |
| *A. curassavica - G. suberosus* | 0.262 | 0.1253 | 90.7 | 2.089 | 0.0979 |

**References**

1. RStudio Team. RStudio: Integrated Development Environment for R. Boston, MA: RStudio, PBC; 2021. Available: http://www.rstudio.com/

2. R Core Team. R: A Language and Environment for Statistical Computing. Vienna, Austria: R Foundation for Statistical Computing; 2021. Available: https://www.R-project.org/

3. Wickham H, Averick M, Bryan J, Chang W, McGowan LD, François R, et al. Welcome to the Tidyverse. Journal of Open Source Software. 2019;4: 1686. doi:10.21105/joss.01686

4. Wilke CO. cowplot: Streamlined Plot Theme and Plot Annotations for “ggplot2.” 2020. Available: https://CRAN.R-project.org/package=cowplot

5. Bates D, Maechler M, Bolker B, Walker S. Fitting Linear Mixed-Effects Models Using lme4. Journal of Statistical Software. 2015;67: 1–48. doi:10.18637/jss.v067.i01.

6. Lenth RV. emmeans: Estimated Marginal Means, aka Least-Squares Means. 2021. Available: https://CRAN.R-project.org/package=emmeans

7. Therneau TM, Grambsch PM. Modeling Survival Data: Extending the Cox Model. New York: Springer; 2000.

8. Kassambara A, Kosinski M, Biecek P. survminer: Drawing Survival Curves using “ggplot2.” 2020. Available: https://CRAN.R-project.org/package=survminer

9. Therneau TM. coxme: Mixed Effects Cox Models. 2020. Available: https://CRAN.R-project.org/package=coxme

10. Hlavac M. stargazer: Well-Formatted Regression and Summary Statistics Tables. Bratislava, Slovakia: Central European Labour Studies Institute (CELSI); 2018. Available: https://CRAN.R-project.org/package=stargazer
